# Synergistic effect of PIP_2_ and PIP_3_ on membrane-induced phase separation of integrin complexes

**DOI:** 10.1016/j.bpj.2025.04.011

**Published:** 2025-04-14

**Authors:** Chiao-Peng Hsu, Arsenii Hordeichyk, Jonas Aretz, Reinhard Fässler, Andreas R. Bausch

**Affiliations:** 1 Heinz Nixdorf Chair for Cellular Biophysics, Center for Functional Protein Assemblies, Center for Organoid Systems, Department of Bioscience, Technical University of Munich, Technical University of Munich School of Natural Sciences, Garching, Germany; 2 Max Planck School Matter to Life, Heidelberg, Germany; 3 Department of Molecular Medicine, Max Planck Institute of Biochemistry, Martinsried, Germany

## Abstract

The assembly of integrin adhesion complexes at the inner leaflet of the plasma membrane regulates cell adhesion to the extracellular matrix. The multivalent protein interactions within the complexes and with the cell membrane display characteristics of membrane-associated biomolecular condensates driven by liquid-liquid phase separation. The composition of lipids and the distribution of the cell membrane are crucial for forming integrin adhesion complexes. Here, we report that phosphatidylinositol 4,5-bisphosphate (${\text{PIP}}_{2}$) and phosphatidylinositol (3,4,5)-trisphosphate (${\text{PIP}}_{3}$) in the model membrane synergistically regulate the formation of membrane-induced integrin adhesion condensates, which consist of β1 tails, kindlin, talin, paxillin, and focal adhesion kinase. We show that the preferential bindings of kindlin to ${\text{PIP}}_{3}$ and talin to ${\text{PIP}}_{2}$ enhance their recruitment to the membrane, which in turn increases the probability of membrane-associated phase separation. Our results indicate that modulating the intricate balance of membrane composition is a strategy to localize integrin adhesion complexes and optimize their density on lipid membranes.

## Significance

Integrin adhesion complexes located at the inner leaflet of the plasma membrane facilitate cell adhesion to extracellular matrix proteins and activate various signaling pathways. The composition and distribution of the lipid membrane affect the formation of integrin adhesion complexes, which are driven by liquid-liquid phase separation. We show that phosphoinositides ${\text{PIP}}_{2}$ and ${\text{PIP}}_{3}$ in the model membrane synergistically govern the formation of membrane-induced integrin adhesion condensates, which consist of β1 tails, kindlin, talin, paxillin, and focal adhesion kinase. Our findings suggest that tuning the membrane composition can effectively localize integrin adhesion complexes and enhance their density on lipid membranes.

## Introduction

Integrin adhesion complexes are essential for cells to sense and respond to the biophysical and biochemical properties of the extracellular matrix, influencing cellular behaviors such as spreading, migration, polarity, differentiation, and proliferation (1,2). Integrins are α-β heterodimers composed of large ectodomains that facilitate ligand binding, single-span transmembrane helices, and short cytoplasmic tails that indirectly link with the contractile actomyosin cytoskeleton (3,4). Upon activation, integrins cluster and form nascent adhesions with early adaptor proteins at the leading edge of migrating cells, whereas the linkage to actin cytoskeleton is critical for the stabilization and maturation of nascent adhesions into focal adhesions (2,5,6). Kindlin, talin, paxillin, and focal adhesion kinase (FAK) are involved in the formation and regulation of nascent adhesions as kindlin glides along the plasma membrane and other proteins diffuse between the cytoplasm and the integrin adhesion complexes (5,7,8,9). The multivalent protein interactions and protein dynamics in the integrin adhesion complexes display characteristics of membrane-associated biomolecular condensates driven by liquid-liquid phase separation (10). In contrast to biomolecular condensates found in the cytoplasm and the nucleus, the spatiotemporal regulation of integrin adhesion complexes is influenced by the composition and organization of the plasma membrane (11,12,13,14). Moreover, cells can benefit from local protein enrichment, which facilitates condensate formation and function in specific locations at membranes. In vitro reconstitutions have shown that phase-separated condensates can emerge at lower concentration thresholds and display slower dynamics when associated with membranes than in bulk solutions (15,16,17).

The composition of the cell membrane, including the types and distribution of lipids and the presence of specific lipid microdomains, is crucial for the formation and regulation of integrin adhesion complexes (12,14,18,19). In particular, the presence of specific phosphoinositides, such as phosphatidylinositol 4,5-bisphosphate (${\text{PIP}}_{2}$) and phosphatidylinositol (3,4,5)-trisphosphate (${\text{PIP}}_{3}$), modulate membrane dynamics and integrin function (20,21). The key adaptor proteins that bind to integrin directly, kindlin and talin, also bind to these phosphoinositides (22). Kindlin binds to ${\text{PIP}}_{2}$ and ${\text{PIP}}_{3}$ via its pleckstrin homology (PH) domain and shows a stronger preference for binding to ${\text{PIP}}_{3}$ than to ${\text{PIP}}_{2}$ (23,24). Talin binds to ${\text{PIP}}_{2}$ via its protein 4.1, ezrin, radixin, moesin domain, which induces a conformational change in talin to expose the integrin-binding site (25). ${\text{PIP}}_{2}$ is typically present in the plasma membrane at a concentration of 1%–3% of the total lipid content, whereas ${\text{PIP}}_{3}$ is present at much lower levels, constituting 0.01%–0.05% of the total lipid content (20,26). Although they comprise only a small fraction of the cell membrane, the balance between ${\text{PIP}}_{2}$ and ${\text{PIP}}_{3}$ in cells is dynamically regulated by a balance of kinase and phosphatase activities to ensure proper cellular function (27). ${\text{PIP}}_{2}$ has been shown to recruit kindlin and talin on the membrane and promote membrane-localized phase separation of nascent adhesions (17). However, the concentration of ${\text{PIP}}_{3}$ can increase at the leading edge of migrating cells due to the activation of phosphoinositide 3-kinase (PI3K) (20,28). How the shift of ${\text{PIP}}_{2}$/PIP_3_ equilibrium influences the membrane-localized phase separation requires further exploration. The colocalization of ${\text{PIP}}_{3}$ enrichment and formation of nascent adhesions at the leading edge of migrating cells prompted us to assess the role of ${\text{PIP}}_{2}$ and ${\text{PIP}}_{3}$ balance in facilitating the phase separation of nascent adhesions.

Here, we demonstrate that kindlin has a preference for binding to ${\text{PIP}}_{3}$, whereas talin has a preference for binding to ${\text{PIP}}_{2}$. These preferences result in enhanced recruitment of kindlin and talin to the membrane, forming complexes with β1 tails. We show that the formation of membrane-induced nascent integrin adhesion condensates, composed of β1 tails, kindlin, talin, paxillin, and FAK, can be tuned by the presence of ${\text{PIP}}_{2}$ and ${\text{PIP}}_{3}$ in the membrane. Our results indicate that tuning the coexistence of ${\text{PIP}}_{2}$ and ${\text{PIP}}_{3}$ synergistically enhances the probability of enabling specific membrane-associated phase separation.

## Materials and methods

### Materials and reagents

Isopropyl-β-D-thiogalactopyranoside, tris, tris(2-carboxyethyl)phosphine (TCEP), hydrogen peroxide (H_2_O_2_), sulfuric acid (H_2_SO_4_), imidazole, sodium hydroxide (NaOH), 4-(2-hydroxyethyl)-1-piperazineethanesulfonic acid (HEPES), potassium chloride (KCl), zinc chloride (ZnCl_2_), sodium azide (NaN_3_), citric acid, ethylenediaminetetraacetic acid (EDTA), Atto 647 NHS-ester, dithiothreitol, glucose, pyranose-oxidase, catalase, ATP, and PI3 kinase (p120γ) were purchased from Sigma-Aldrich (St. Louis, Missouri). 1,2-dioleoyl-sn-glycero-3-phosphocholine (DOPC), 1,2-dioleoyl-sn-glycero-3-[(N-(5-amino-1-carboxypentyl)iminodiacetic acid)succinyl] (nickel salt) (DGS-NTA-Ni), 1,2-dioleoyl-sn-glycero-3-phosphoethanolamine-N-[methoxy(polyethylene glycol)-2000] (ammonium salt) (PEG2000-PE), 1,2-dioleoyl-sn-glycero-3-phospho-(1′-myo-inositol-4′,5′-bisphosphate) (ammonium salt) (PI(4,5)P_2_), and 1,2-dioleoyl-sn-glycero-3-phospho-(1′-myo-inositol-3′,4′,5′-trisphosphate) (ammonium salt) (PI(3,4,5)P_3_) were purchased from Avanti Polar Lipids (Alabaster, Alabama). 1,2-dihexadecanoyl-sn-glycerin-3-phosphoethanolamin (Oregon Green DHPE) was purchased from Thermo Fisher Scientific (Waltham, Massachusetts).

### Buffers

HEPES buffer (pH 7.4) contains 50 mM HEPES, 1 mM dithiothreitol, 2.5 μM ZnCl_2_, and 150 mM KCl. The buffer contains 2.5 μM ${\text{ZnCl}}_{2}$ to ensure the binding of kindlin and paxillin (9,29,30). Citric buffer (pH 4.85) contains 20 mM citric acid, 50 mM KCl, 0.1 mM EDTA, and 0.1 mM NaN_3_.

### Protein expression and purification

Atto643-labeled ${\text{his}}_{6}$-β1 integrin cytoplasmic tail peptides were synthesized and purified by the Max Planck Institute of Biochemistry (MPIB) Core Facility. The correct peptide sequence was controlled by high-resolution intact mass spectrometry.

Kindlin-2 and paxillin were expressed and purified as described earlier (29). Briefly, an N-terminal his-sumo tag was added to express the proteins soluble in *E. coli* (DE3) Rosetta 2. After cell lysis, the proteins were captured with a Ni-NTA-immobilized metal ion affinity chromatography (IMAC); the sumo tag was removed by SenP2 digest and subsequent pull-down with Ni-NTA beads followed by a final size-exclusion chromatography.

His-tagged talin-1 was expressed and purified as described earlier (31) with minor changes. The protein was expressed in *E. coli* (DE3) Rosetta 2 upon induction with 0.2 mM isopropyl-β-D-thiogalactopyranoside overnight at 18°C. The cells were harvested by centrifugation and lysed by sonication in IMAC running buffer (25 mM Tris [pH 7.8], 500 mM NaCl, and 1 mM TCEP) supplemented with 2 mM ${\text{MgCl}}_{2}$, 40μ L DNaseI (obtained from the MPIB Core Facility), and a spatula tip of lysozyme. After clearing the lysate, the sample was loaded on a 5 mL HisTrap HP Ni-NTA IMAC column (17524801; Cytiva, Marlborough, Massachusetts), washed with IMAC running buffer, and eluted with a stepwise gradient with IMAC elution buffer (25 mM Tris [pH 7.8], 500 mM NaCl, 1 mM TCEP, and 500 mM imidazole). Elution fractions were pooled, concentrated, and further purified with a Superose 6 Increase 10/300 GL size-exclusion chromatograph (29091596, Cytiva) using 20 mM Tris (pH 7.8), 200 mM NaCl, and 1 mM TCEP as running buffer.

Murine FAK carrying an N-terminal his-sumo tag was cloned using Gateway cloning into PB-T-Rfa to establish a stable FAK-expressing HEK293T cell line (32). The cells were cultured in FreeStyle 293 Expression Medium (12338018, Gibco, Waltham, Massachusetts) until reaching a cell density of $10^{6}$ cells per mL before inducing protein expression by doxycycline addition (1 μg/mL) for 3 days. Cells were collected by centrifugation, resuspended in IMAC running buffer (25 mM Tris [pH 7.5], 500 mM NaCl, 10% glycerol, and 1 mM TCEP) supplemented with a cOmplete, EDTA-free protease inhibitor tablet and 40 μL benzonase (obtained from the MPIB Core Facility) and lysed by douncing on ice. Centrifugation-cleared lysate (60 min, 58,000*g*, 4°C) was sterile filtered, applied to Ni-NTA IMAC column (HisTrap SP, 5 mL, Cytiva), washed with IMAC running buffer, and eluted with a stepwise gradient with IMAC elution buffer (25 mM Tris [pH 7.5], 500 mM imidazole, 500 mM NaCl, 10% glycerol, and 1 mM TCEP). The protein was diafiltered using a 30 kDa cutoff Amicon Ultra 15 (UFC903024; Merck Millipore, Livingston, Scotland) filter against IMAC running buffer, and the his-sumo-tag was cleaved using sumo protease (obtained from the MPIB Core Facility) overnight at 4°C. The cleaved protein was further purified with size-exclusion chromatography to remove any protein aggregates using a Superdex 200 Increase 10/300 GL column (28-9909-44; Cytiva) in phosphate-buffered saline supplemented with additional 150 mM NaCl and 1 mM TCEP.

All protein preparations were controlled for their integrity and purity using SDS-PAGE, high-resolution intact mass spectrometry, UV-visible spectrum, and dynamic light scattering (Figs. S1 and S2).

### Small unilamellar vesicle production

Lipid mixtures of 5% ${\text{PIP}}_{2}$ (89.3 mol % DOPC, 5 mol % DGS-NTA-Ni, 5 mol % PI(4,5)P_2_, 0.5 mol % PEG2000-PE, and 0.2 mol % Oregon Green DHPE), 5% ${\text{PIP}}_{3}$ (89.3 mol % DOPC, 5 mol % DGS-NTA-Ni, 5 mol % PI(3,4,5)P_3_, 0.5 mol % PEG2000-PE, and 0.2 mol % Oregon Green DHPE), and 2.5% ${\text{PIP}}_{2}$ + 2.5% ${\text{PIP}}_{3}$ (89.3 mol % DOPC, 5 mol % DGS-NTA-Ni, 2.5 mol % PI(4,5)P_2_, 2.5 mol % PI(3,4,5)P_3_, 0.5 mol % PEG2000-PE, and 0.2 mol % Oregon Green DHPE) were mixed in chloroform. Lipid films were prepared by drying the lipid mixtures under a stream of nitrogen and placed under a vacuum for at least 2 h. Lipid films were hydrated and resuspended by sonication for 30 min in citric buffer at a final lipid concentration of 0.5 mM. Small unilamellar vesicles (SUVs) were then created by extruding lipid suspensions 20 times through 100-nm-pore membrane filters (Whatman) using a mini-extruder (Avanti Polar Lipids). SUVs were stored at 4°C and used within 72 h.

### Flow chamber preparation

Flow chambers (≈40 μL) that consist of coverslips (22 × 22 mm; Karlsruhe, Germany, Carl Roth) fixed to microscope slides (25 × 75 mm; Carl Roth) by three-layer parafilm were used for the assays. The coverslips were sonicated for 30 min in 3 M NaOH and rinsed with MilliQ $H_{2}$O before being cleaned in piranha solution (2:1, H_2_SO_4_/H_2_O_2_) for 10 min to render the surface hydrophilic. The piranha-cleaned coverslips were then rinsed and stored in MilliQ $H_{2}$O. The microscope slides were sonicated for 30 min in 2 wt % Hellmanex aqueous solution (Hellma, Plainview, New York) and rinsed with MilliQ $H_{2}$O before being stored in ethanol. The coverslips and microscope slides were used within 72 h after cleaning.

### Model membrane system reconstitution

Supported lipid bilayers (SLBs) were prepared in flow chambers. SUVs were added to chambers at a final lipid concentration of 0.167 mM and allowed to rupture and form an SLB on the glass surface for 20 min at room temperature. Afterward, the SLBs were first washed with 0.8 mL citric buffer (33) and then 0.8 mL HEPES buffer to remove excess liposomes. SLBs were incubated with 1 μM ${\text{his}}_{6}$-tagged integrin β1 tails in HEPES buffer for 20 min and then washed with 0.8 mL HEPES buffer. The β1-bound SLBs were incubated with different protein mixtures. Assays with p120γ contain 2 mM ATP to ensure kinase activity. The protein mixtures contain 8 U/mL pyranose-oxidase. Depending on the assays, the 7 kU/mL catalase and 36 mM glucose function as an oxygen-scavenging system to prevent protein denature and photobleaching during fluorescence imaging.

### Imaging and data acquisition

A Leica DMi8 microscope with an HC PL APO 100×/1.47 oil immersion objective was used to perform the fluorescence imaging using an ORCA-Flash 4.0 CMOS camera (C13440-20CU; Hamamatsu, Shizuoka, Japan). A Leica Infinity Scanner unit was used to perform fluorescence recovery after photobleaching (FRAP) experiments. Fiji/ImageJ was used to analyze the density of β1 clusters (34).

### FRAP experiments

A circle with a diameter of 5 μm was bleached with a 638 nm laser, and fluorescence images were acquired for 80 s. A region of the SLB outside the circle was used for background subtraction. Fluorescence intensity values within bleached regions were exported using LAS X software. Data were normalized to prebleach levels and fitted to a double-component exponential recovery function, $F\left(t\right)=y_{0}+A_{fast}\cdot e^{-t/\tau_{fast}}+A_{slow}\cdot e^{-t/\tau_{slow}}$, where F(t) is the relative fluorescence intensity over time, $\frac{-\left(A_{fast}+A_{slow}\right)}{1-\left(y_{0}+A_{fast}+A_{slow}\right)}$ is the mobile fraction, $\tau_{fast}$ is the fast recovery time constant, and $\tau_{slow}$ is the slow recovery time constant. $t_{1/2\_fast}=\tau_{fast}\cdot \ln\;2$ and $t_{1/2\_slow}=\tau_{slow}\cdot \ln\;2$ are the fast and slow half-recovery times, respectively. Fitting was performed using Python3.

## Results

### PIP_2_/PIP_3_-dependent slowdown of β1 diffusion in the presence of kindlin and talin

**Figure 1:**
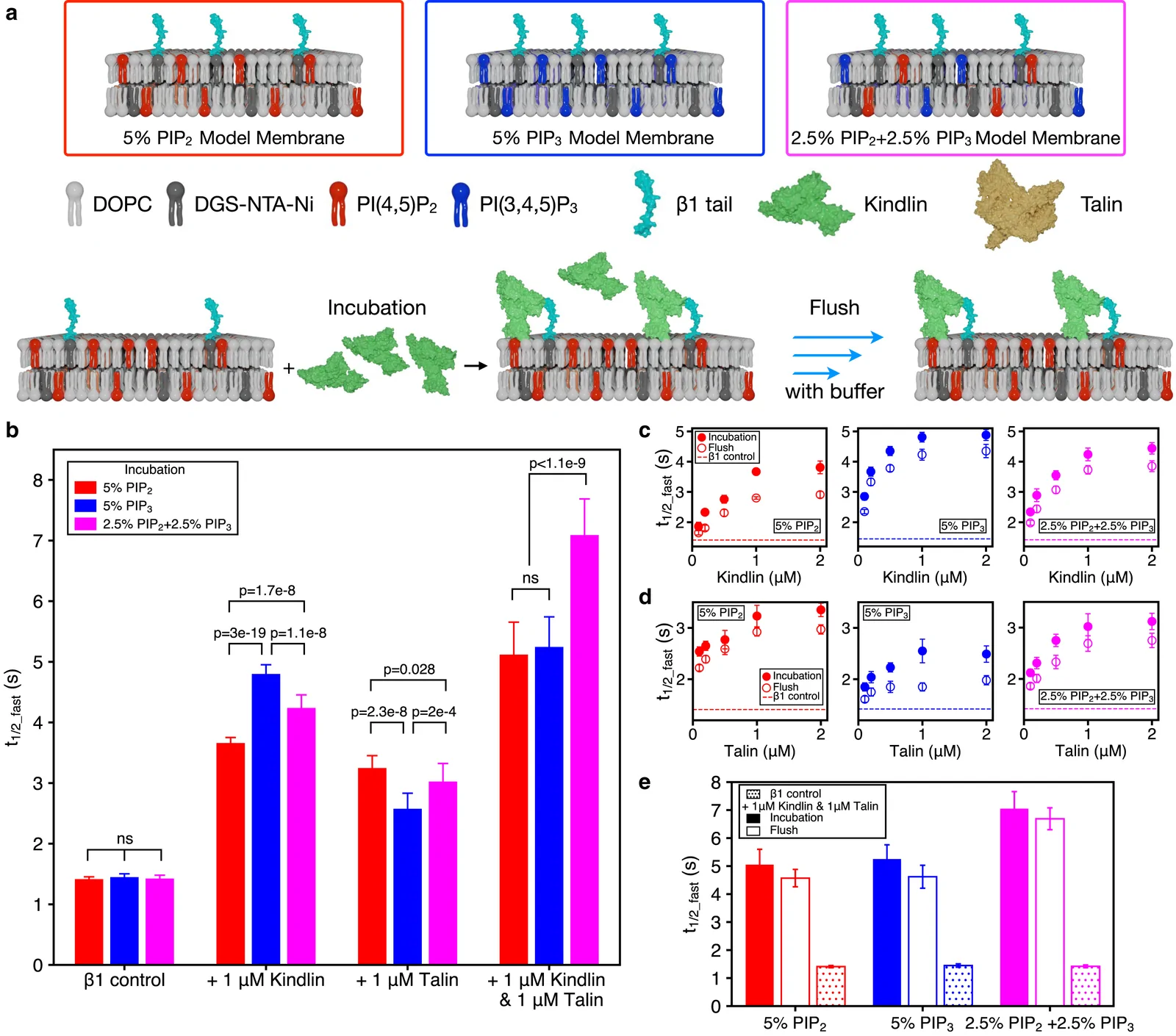
Diffusion of β1 integrin tails on ${\text{PIP}}_{2}$/PIP_3_ membranes. (*a*) Schematic of the reconstituted model membranes containing 5% ${\text{PIP}}_{2}$, 5% ${\text{PIP}}_{3}$, and 2.5% ${\text{PIP}}_{2}$ + 2.5% ${\text{PIP}}_{2}$, as well as the incubation and flush experiment steps with proteins. (*b*) Diffusion of $\beta 1\;\left(t_{1/2\_fast}\right)$ after 30-min incubation with kindlin/talin on 5% ${\text{PIP}}_{2}$, 5% ${\text{PIP}}_{3}$, and 2.5% ${\text{PIP}}_{2}$ + 2.5% ${\text{PIP}}_{2}$ membranes. Significance was tested with an unpaired *t*-test. (*c* and *d*) Diffusion of β1as a function of kindlin (*c*) or talin (*d*) concentration after 30-min incubation and after flush on 5% ${\text{PIP}}_{2}$ (*left*), 5% ${\text{PIP}}_{3}$ (*middle*), and 2.5% ${\text{PIP}}_{2}$ + 2.5% ${\text{PIP}}_{2}$ (*right*) membranes. (*e*) Diffusion of β1after 30-min incubation with 1 μM kindlin and 1 μM talin and after flush on 5% ${\text{PIP}}_{2}$, 5% ${\text{PIP}}_{3}$, and 2.5% ${\text{PIP}}_{2}$ + 2.5% ${\text{PIP}}_{2}$ membranes. Error bars in (*b*)–(*e*) represent the standard deviations of 15 measurements from 3 independent experiments.

To study how phosphoinositides affect the binding of β1 tails to kindlin and talin on membranes, we reconstitute 5 mol % ${\text{PIP}}_{2}$, 5 mol % ${\text{PIP}}_{3}$, and 2.5 mol % ${\text{PIP}}_{2}$ + 2.5 mol % ${\text{PIP}}_{3}$ SLB membranes with attached β1 tails (materials and methods; Fig. 1 *A*). The diffusion of uniformly distributed β1 tails on the model membranes reflects the interaction between β1 tails, kindlin, talin, and the membrane (17,35). The diffusion of tethered β1 is correlated with its hydrodynamic radius on the membrane. The binding of β1 to proteins or lipids can result in the formation of transient and stable complexes on the membrane, which possess a larger hydrodynamic radius than a tethered β1. Thus, the measurement of β1 diffusion indicates the interactions with other components on the membrane. The diffusion of β1 tails is determined by FRAP measurements, enabling the calculation of the half-recovery time. A longer half-recovery time corresponds to slower diffusion of the β1 tails on the model membranes (materials and methods; Figs. S3 and S4). The diffusion of uniformly distributed β1 tails on the membrane depends on the incubated kindlin and talin as well as ${\text{PIP}}_{2}$ and ${\text{PIP}}_{3}$ (Fig. 1 *B*). Without kindlin and talin, β1 tail diffusion remains unaffected by ${\text{PIP}}_{2}$ and ${\text{PIP}}_{3}$, excluding a potential interaction of the β1 tail with the phosphoinositides. Although the incubation of 1 μM kindlin or 1 μM talin reduces the diffusion of β1 tails on the lipid membranes, the reduction of β1diffusibility varies within the three model membranes (Fig. 1 *B*).

The slowdown of β1 tail diffusion in the presence of 1 μM kindlin is enhanced on 5% ${\text{PIP}}_{3}$ membranes compared to 5% ${\text{PIP}}_{2}$ membranes. However, the effect is reversed in the presence of 1 μM talin (Fig. 1 *B*). The diffusion of β1 tails is 2.6 and 3.3 times slower when incubated with 1 μM kindlin on 5% ${\text{PIP}}_{2}$ and 5% ${\text{PIP}}_{3}$ membranes, respectively, compared to β1 tails alone. Conversely, when incubated with 1 μM talin, the diffusion of β1 tails is 2.3 and 1.8 times slower on 5% ${\text{PIP}}_{2}$ and 5% ${\text{PIP}}_{3}$ membranes, respectively, compared to β1 tails alone. The ${\text{PIP}}_{2}$/PIP_3_-dependent slowdown of β1 tail diffusion on the membrane is the consequence of kindlin and talin binding to the phosphoinositides in lipid membranes via their PH and protein 4.1, ezrin, radixin, moesin domains, respectively (22,23,24,25). Although it has been identified that kindlin prefers to bind ${\text{PIP}}_{3}$ (23,24) and talin binds to ${\text{PIP}}_{2}$ (22,25), we perform cosedimentation assays to confirm the binding between kindlin/talin and ${\text{PIP}}_{2}$/PIP_3_ lipids used in this study (Fig. S5). In addition, these preferential interactions between kindlin, talin, and phosphoinositides have been observed with reconstituted giant unilamellar vesicles (36).

On 2.5% ${\text{PIP}}_{2}$ + 2.5% ${\text{PIP}}_{3}$ membranes, this slowdown caused by 1 μM kindlin or 1 μM talin incubation falls between the values observed for 5% ${\text{PIP}}_{2}$ and 5% ${\text{PIP}}_{3}$ membranes. Yet, the most significant reduction of β1 tail diffusion is observed on mixed phosphoinositide membranes when incubating kindlin and talin together instead of separately. Upon incubation of 1 μM kindlin and 1 μM talin, β1 tails diffuse 3.6 times slower on 5% ${\text{PIP}}_{2}$ and 5% ${\text{PIP}}_{3}$ membranes and 4.9 times slower on 2.5% ${\text{PIP}}_{2}$ + 2.5% ${\text{PIP}}_{3}$ membranes. The reduction in diffusibility and mobile fraction of β1 tails on these three membranes (Fig. 1 *B*; Fig. S1) suggests that ternary β1-kindlin-talin complexes form on these membranes.

Kindlin and talin are recruited to the lipid membrane due to the presence of phosphoinositides, which effectively enhances their local concentration and binding probability to β1 tails. To further evaluate the off-rate of kindlin (Fig. 1 *C*) and talin (Fig. 1 *D*) to β1 tails on phosphoinositide membranes, we compare the diffusion of β1 tails after a 30-min incubation and then after flushing with buffer. In these experiments, 0.1–2 μM of kindlin or talin are incubated individually with the three model membranes. On all three phosphoinositide membranes, the diffusion of β1 tails after flushing decreases slightly and is slower than the diffusion of β1 tails alone. This indicates that kindlin, as well as talin alone, can already form stable complexes with β1on the 5% ${\text{PIP}}_{2}$, 5% ${\text{PIP}}_{3}$, and 2.5% ${\text{PIP}}_{2}$ + 2.5% ${\text{PIP}}_{3}$ membranes. Although kindlin shows a stronger preference for binding ${\text{PIP}}_{3}$ compared to ${\text{PIP}}_{2}$, talin has the opposite preference. On 5% ${\text{PIP}}_{3}$ membranes, all diffusion times of β1incubated with kindlin are longer than on 5% ${\text{PIP}}_{2}$ membranes. In contrast, all diffusion times of β1incubated with talin are longer on 5% ${\text{PIP}}_{2}$ membranes than on 5% ${\text{PIP}}_{3}$ membranes.

Despite the different preferences of kindlin and talin on ${\text{PIP}}_{2}$ and ${\text{PIP}}_{3}$, the 2.5% ${\text{PIP}}_{2}$ + 2.5% ${\text{PIP}}_{3}$ membranes effectively recruit these proteins (Figs. 1, *C* and *D*). The simultaneous incubation with kindlin and talin leads to larger complexes with β1 tails in the presence of phosphoinositide, as shown by the increased diffusion time after flushing (Fig. 1 *E*). These complexes lead to 3.2 times slower diffusion of β1 tails compared to the diffusion of β1 tails in the absence of kindlin or talin on 5% ${\text{PIP}}_{2}$ and 5% ${\text{PIP}}_{3}$ membranes. Synergistically, on mixed phosphoinositide membranes, the β1 tails diffuse 4.7 times slower in the same condition, indicating more β1-kindlin-talin complex formation on the membranes. The formation of β1-kindlin-talin complexes on these model membranes prompts us to explore how the phosphoinositide membrane can induce the recruitment of paxillin and FAK from the solution to the membrane to form minimal integrin adhesion complexes (17,37).

### PIP_2_ and ${\text{PIP}}_{3}$ enhance the emergence of β1 clusters on membranes

**Figure 2:**
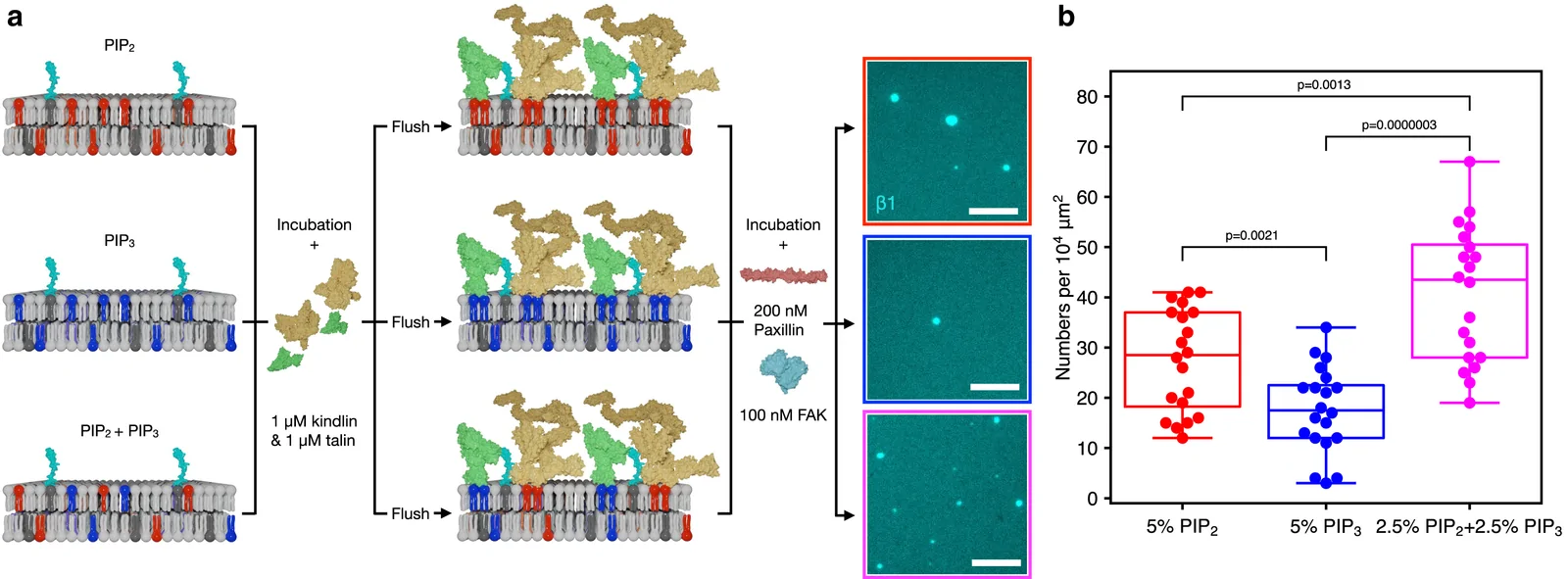
PIP_2_/PIP_3_ membrane synergistically enhances the emergence of β1 clusters. (*a*) Sequential incubation procedure leads to the emergence of β1 clusters after 90-min incubation on 5% ${\text{PIP}}_{2}$, 5% ${\text{PIP}}_{3}$, and 2.5% ${\text{PIP}}_{2}$ + 2.5% ${\text{PIP}}_{2}$ membranes. Scale bars, 10 μm. (*b*) The density of β1 clusters formed after 90-min incubation with the three membranes in (*a*). Each condition contains 20 measurements from 4 independent experiments. Boxplots indicate the median (*line*), 25th and 75th percentile (*box*), and 0th and 100th percentile (*whiskers*). Significance was tested with an unpaired *t*-test.

It has been shown that the sequential assembly of kindlin and talin followed by paxillin and FAK can generate surface-induced condensate formation, which leads to the clustering of β1 tails on ${\text{PIP}}_{2}$ membranes (17). To determine whether the condensate formation depends on different phosphoinositides in the membranes, we reconstitute the β1-kindlin-talin complexes on 5% ${\text{PIP}}_{2}$, 5% ${\text{PIP}}_{3}$, and 2.5% ${\text{PIP}}_{2}$ + 2.5% ${\text{PIP}}_{3}$ membranes via incubation and after the flushing steps (Fig. 2 *A*). After the incubation of 200 nM paxillin and 100 nM FAK at a high ionic strength of 150 mM KCl, mimicking physiological conditions, β1 tail clusters emerge spontaneously on all phosphoinositide membranes (Fig. 2 *A*). The colocalization of β1and kindlin indicates the partitioning of kindlin in these surface-induced condensates, and FRAP measurements demonstrate that β1 clusters are dynamic assemblies where β1 tails diffuse freely and exchange on the lipid membrane (Fig. S6). The emergence of β1 tail clusters suggests that the local biochemical environment of the membranes induces the phase separation of paxillin and FAK. Yet, the density and area fraction of β1 clusters depend on the phosphoinositides in the membranes (Fig. 2 *B*; Fig. S7). The β1cluster density on 2.5% ${\text{PIP}}_{2}$ + 2.5% ${\text{PIP}}_{3}$ membranes increases 1.5- and 2-fold compared to the ones on 5% ${\text{PIP}}_{2}$ and 5% ${\text{PIP}}_{3}$ membranes, respectively (Fig. 2 *B*). Our observations show that the mix of ${\text{PIP}}_{2}$ and ${\text{PIP}}_{3}$ in the lipid membrane optimizes the local environment to increase the probability of surface-induced phase separation. Moreover, the intricate balance of ${\text{PIP}}_{2}$ and ${\text{PIP}}_{3}$ in the cell membranes motivates us to investigate how the conversion of ${\text{PIP}}_{2}$ and ${\text{PIP}}_{3}$ impacts the emergence of β1 clusters.

### Balance of ${\text{PIP}}_{2}$ and ${\text{PIP}}_{3}$ regulates the emergence of β1 clusters on membranes

**Figure 3:**
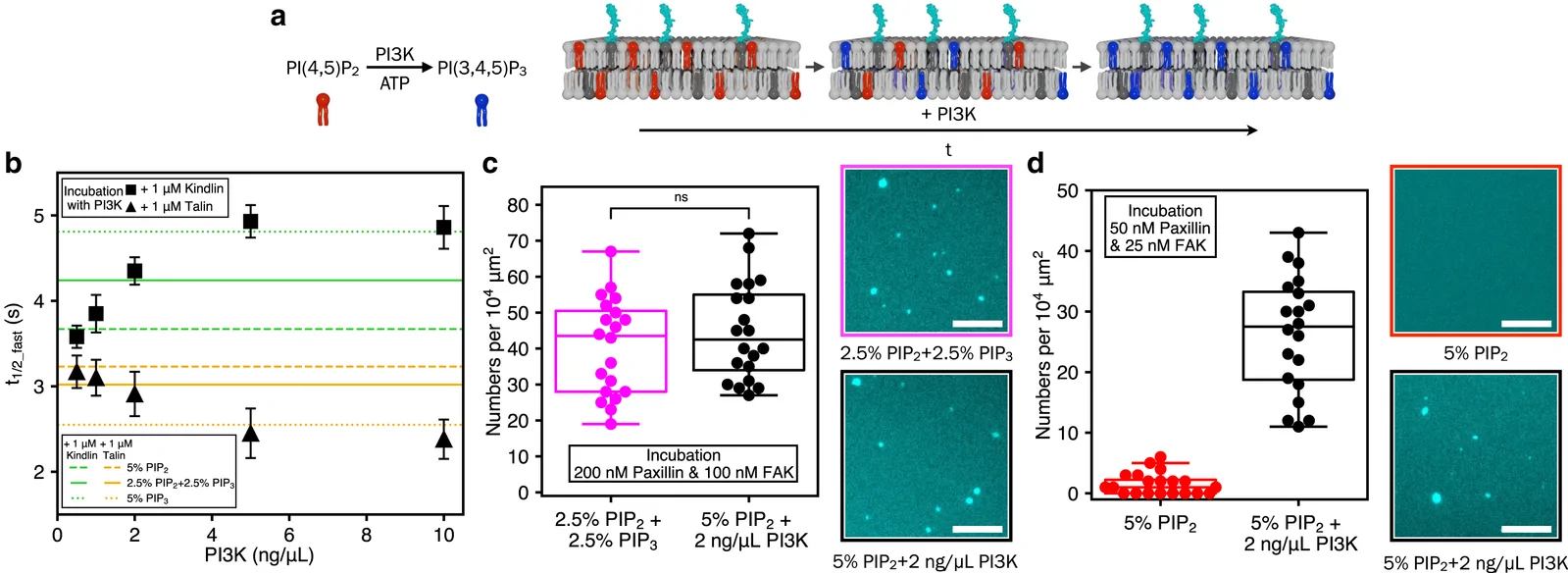
PI3 kinase modifies the membrane surfaces and regulates the emergence of β1 clusters. (*a*) Schematic of PI3 kinase catalyzes the conversion of PI(4,5)P_2_ into PI(3,4,5)P_3_. (*b*) Diffusion of β1as a function PI3 kinase concentration after 30-min incubation with kindlin or talin. (*c*) The density (*left*) and images (*right*) of β1 clusters formed after 90-min sequential incubation (200 nM paxillin and 100 nM FAK) on 2.5% ${\text{PIP}}_{2}$ + 2.5% ${\text{PIP}}_{2}$ membranes (same data as Fig. 2*B*) and 5% ${\text{PIP}}_{2}$ membranes with 2 ng/μL PI3K. (*d*) The density (*left*) and images (*right*) of β1 clusters formed after 90-min sequential incubation (50 nM paxillin and 25 nM FAK) on 5% ${\text{PIP}}_{2}$ membranes and 5% ${\text{PIP}}_{2}$ membranes with 2 ng/μL PI3K. (*c* and *d*) Each condition contains 20 measurements from 4 independent experiments. Boxplots indicate the median (*line*), 25th and 75th percentile (*box*), and 0th and 100th percentile (*whiskers*). Significance was tested with an unpaired *t*-test. Scale bars, 10 μm.

The balance between ${\text{PIP}}_{2}$ and ${\text{PIP}}_{3}$ in cells is tightly regulated by PI3K together with phosphatase and tensin homolog (PTEN) (27). PI3K converts ${\text{PIP}}_{2}$ to ${\text{PIP}}_{3}$ by adding a phosphate group at the 3-position, and PTEN converts ${\text{PIP}}_{3}$ back to ${\text{PIP}}_{2}$ by removing the 3-phosphate. Therefore, ${\text{PIP}}_{2}$ model membranes can be transformed to ${\text{PIP}}_{3}$ model membranes with PI3K (Fig. 3 *A*). We measure the diffusion of β1 tails with 1 μM of kindlin or talin and 0.5–10 ng/μL of PI3K after 30-min incubation on 5% ${\text{PIP}}_{2}$ membranes (Fig. 3 *B*). At low concentrations of PI3K (0.5 ng/μL), the diffusion of β1 tails is close to that measured on 5% ${\text{PIP}}_{2}$ membranes (Fig. 1 *B*). With increasing concentrations of PI3K, the diffusion of β1 tails is either slower or faster depending on the incubation with either kindlin or talin. At above 5 ng/μL of PI3K, the diffusion of β1 tails resembles that measured on 5% ${\text{PIP}}_{3}$ membranes (Fig. 1 *B*). The change of β1 tail diffusibility with the incubation of kindlin or talin indicates the transformation of ${\text{PIP}}_{2}$ membranes to ${\text{PIP}}_{3}$ membranes. Particularly, at 2 ng/μL of PI3K, the measured fast recovery times suggest the equal-molarity coexistence of ${\text{PIP}}_{2}$ and ${\text{PIP}}_{3}$ in the model membranes, enabled by the kinase activity. We then perform sequential assembly experiments where 2 ng/μL of PI3K is added to 1 μM kindlin and 1 μM talin on 5% ${\text{PIP}}_{2}$ membranes. The β1 tail clusters emerge after the addition of 200 nM paxillin and 100 nM FAK, and the density and area fraction are comparable with the ones that emerge on 2.5% ${\text{PIP}}_{2}$ + 2.5% ${\text{PIP}}_{3}$ membranes (Fig. 3 *C*; Fig. S8). Moreover, at lower concentrations of paxillin and FAK (50 nM paxillin and 25 nM FAK), barely any β1 clusters form on the 5% ${\text{PIP}}_{2}$ membrane, but β1 clusters can be observed on the 5% ${\text{PIP}}_{2}$ membrane with 2 ng/μL PI3K (Fig. 3 *D*; Fig. S9). This observation demonstrates that the addition of PI3K shifts the phase separation boundary of the membrane-bound condensate system. These results indicate that the balance of ${\text{PIP}}_{2}$ and ${\text{PIP}}_{3}$ plays a key role in promoting the formation of membrane-associated nascent integrin adhesion condensates. The presence of both ${\text{PIP}}_{2}$ and ${\text{PIP}}_{3}$ enhances the probability of surface-induced phase separation.

## Discussion

**Figure 4:**
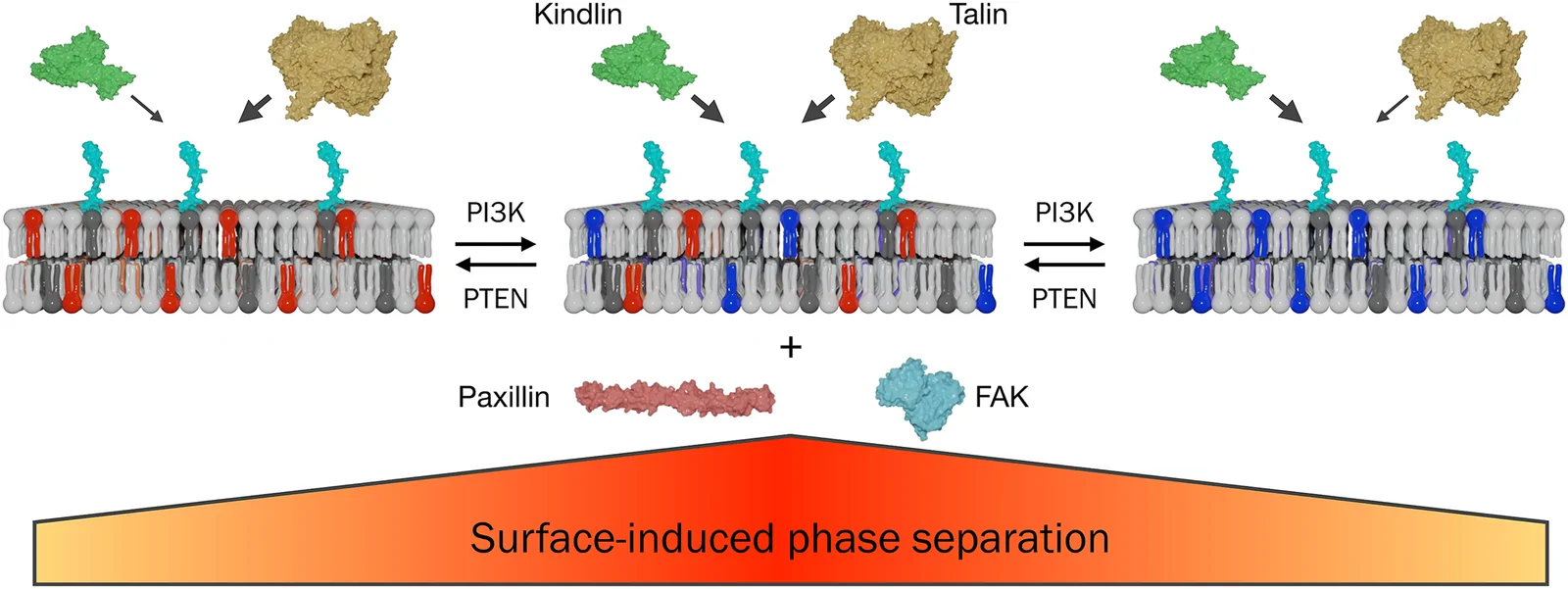
PIP_2_/PIP_3_ cycle sets membrane surfaces to steer the phase separation. Schematic of the balance between ${\text{PIP}}_{2}$ and ${\text{PIP}}_{3}$ optimizes the surface-induced phase separation.

In the present paper, we show that the balance of ${\text{PIP}}_{2}$ and ${\text{PIP}}_{3}$ determines the density of nascent integrin clusters on lipid membranes under surface-induced phase separation conditions. The slowdown of β1 tail diffusion on the lipid membrane indicates that kindlin prefers to bind ${\text{PIP}}_{3}$, and talin prefers to bind ${\text{PIP}}_{2}$. Although the complex formation of kindlin, talin, and β1 tails (38) on ${\text{PIP}}_{2}$- and ${\text{PIP}}_{3}$-only membranes can induce the recruitment and phase separation of paxillin and FAK, the highest probability of finding nascent integrin clusters is observed on mixed phosphoinositide model membranes with the same protein concentrations and assembling sequence. This synergistic observation is confirmed by shifting the ${\text{PIP}}_{2}$/${\text{PIP}}_{3}$ equilibrium in the lipid membrane via PI3K. Thus, the balance of ${\text{PIP}}_{2}$ and ${\text{PIP}}_{3}$, regulated by PI3K and PTEN, in the lipid membrane is crucial for the effective localization and assembly of reconstituted minimal adhesion complexes (Fig. 4). Although ${\text{PIP}}_{2}$ alone can lower the concentration thresholds for initiating the adhesion complex via surface-induced phase separation (17), our results suggest that ${\text{PIP}}_{3}$ acts as an additional tuning knob to increase the probability of forming nascent adhesion sites. Unlike bulk condensates wetting the membranes (39), the surface-induced membrane-bound condensates resemble prewetting transitions on the surface (40,41). In prewetting transitions, a bulk phase below its saturation concentration is stabilized through favorable interactions with a surface, leading to the formation of a surface film. Moreover, below the saturation concentration, surface binding modulates bound and unbound molecules near the surface, resulting in various surface states and complex surface phase diagrams (42,43). With fixed protein concentrations in bulk, having two regulating knobs on the membrane, ${\text{PIP}}_{2}$ and ${\text{PIP}}_{3}$, potentially serves as a strategy to localize adhesion sites and optimize their density on lipid membranes. The ratio of ${\text{PIP}}_{2}$ and ${\text{PIP}}_{3}$ determines the levels of kindlin and talin on the membrane, which sets the kindlin/talin ratio (Fig. 4). The ratio of kindlin/talin in nascent adhesion is 2:1 and becomes 1:1 when adhesion sites start to mature (5). The imbalance of ${\text{PIP}}_{2}$ and ${\text{PIP}}_{3}$ can disrupt kindlin-talin cooperativity and lead to the disassembly of focal adhesions (38). To obtain insights on how protein condensates couple with the membranes (44), several in vitro studies have investigated the phase separation of focal adhesion proteins on SLBs. Kindlin, paxillin, and FAK have been shown to spontaneously phase separate in bulk and be bound by integrin tails to phosphoinositides-free membranes (45). Furthermore, ${\text{PIP}}_{2}$-containing membranes trigger the phase separation of focal adhesion proteins on the membrane surface, capturing the formation of nascent adhesions with low protein concentrations (17,46). Our model membrane reconstitutions highlight the importance of tuning the membrane compositions to form and regulate integrin adhesion complexes.

The diversity of membrane lipid composition is key to the various signaling pathways and cell functions (47). In cells, the phosphoinositide binding motifs in kindlin and talin are essential for assembling focal adhesion sites (13,48). Although the plasma membrane comprises only a small fraction of ${\text{PIP}}_{2}$ and ${\text{PIP}}_{3}$, the conversion of ${\text{PIP}}_{2}$ to ${\text{PIP}}_{3}$ via PI3K is crucial for cell signaling and migration (49). We show that the density of minimal adhesion complexes can be maximized on PI3K-modulated mixed phosphoinositides membranes. In *Dictyostelium*, the spatial restriction of ${\text{PIP}}_{3}$ at the leading edge is responsible for cell migration, and the lack of PI3Ks reduces the migration efficiency (50). The redistribution of ${\text{PIP}}_{3}$ at the cell front, using optical control, has been shown to initiate immune cell migration (51). Although ${\text{PIP}}_{3}$ facilitates the formation of membrane-induced nascent adhesion complexes compared to ${\text{PIP}}_{2}$-only membranes, its role in localizing and activating other signaling proteins involved in adhesion maturation is equally significant. ${\text{PIP}}_{3}$ serves as a docking site for proteins with PH domains, such as 3-phosphoinositide-dependent protein kinase-1 (PDK1) and protein kinase B (AKT). PDK1 and AKT are essential for the signaling pathways that regulate focal adhesion dynamics and cytoskeletal organization (52,53). Further reconstitutions, including the linkage to actomyosin and signaling proteins, need to be examined in future studies to identify the role of ${\text{PIP}}_{3}$ in developing focal adhesions. In addition to phosphoinositides, other plasma membrane compositions modulate cell adhesion dynamics, such as cholesterol and sphingolipids (54,55). Lipid domains enriched in cholesterol and sphingolipids can concentrate integrins and other focal adhesion proteins, facilitating their clustering and promoting the assembly and function of focal adhesions. How different lipid membranes orchestrate to form adhesion complexes can be explored with the membrane assay presented in this study.

By influencing the localization, conformation, and activity of membrane proteins, lipids play a critical role in maintaining cellular equilibrium and responding to environmental cues (21). This study sets a framework for understanding how complex lipids synergistically modulate the formation of adhesion complexes. Understanding further protein-membrane interactions provides insights into the molecular mechanisms underlying many physiological processes and diseases governed by cell adhesions.

## Acknowledgments

We dedicate this work to the memory of Erich Sackmann, whose teaching, mentorship, and friendship profoundly influenced our research. His unwavering support, insightful guidance, and dedication to biophysics and science have been instrumental in shaping our research.

This paper stands as a testament to his lasting impact, and his legacy will continue to inspire and guide future endeavors.

We gratefully acknowledge financial support from the 10.13039/501100000781European Research Council (ERC) under the European Union’s Horizon 2020 research and innovation programme (grant agreement no. 810104-PoInt). This research was conducted within the Max Planck School Matter to Life, supported by the German 10.13039/501100002347Federal Ministry of Education and Research (BMBF) in collaboration with the 10.13039/501100004189Max Planck Society.

## Author contributions

C.-P.H., R.F., and A.R.B. designed the research; C.-P.H., A.H., and J.A. performed the research; C.-P.H. and A.H. analyzed the data; and C.-P.H. and A.R.B. wrote the manuscript. All authors reviewed and revised the manuscript.

## Declaration of interests

The authors declare no competing interests.

## Supporting material

Document S1. Figures S1–S9 and supporting material

Document S2. Article plus supporting material
