# Supplementary material for: Synergistic effect of PIP2 and PIP3 on membrane-induced phase separation of integrin complexes
Source: Biophys J. 2025 Apr 14;125(12):2936–44. doi: 10.1016/j.bpj.2025.04.011 (PMC13507320; doi:10.1016/j.bpj.2025.04.011)
Supplement: Supplementary file 1 [file mmc1.pdf]

Biophysical Journal, Volume *125*

Supplemental information

Synergistic effect of PIP_2 and PIP_3 on membrane-induced phase separation of integrin complexes

Chiao-Peng Hsu, Arsenii Hordeichyk, Jonas Aretz, Reinhard Fässler, and Andreas R. Bausch

Supplementary Material for Synergistic effect of PIP_2 and PIP_3 on membrane-induced phase separation of integrin complexes

Chiao-Peng Hsu,[1] Arsenii Hordeichyk,[1,2] Jonas Aretz,[3] Reinhard Fssler,[3] and Andreas R. Bausch[1,2,*]

[1] *Chair for Cellular Biophysics, Center for Functional Protein Assemblies, Center for Organoid Systems, Department of Bioscience, Technical University of Munich, Technical University of Munich School of Natural Sciences, Garching 85748, Germany*

[2] *Max Planck School Matter to Life, Jahnstrae 29, D-69120 Heidelberg, Germany*

[3] *Department of Molecular Medicine, Max Planck Institute of Biochemistry, Martinsried 82152, Germany*

CO-SEDIMENTATION ASSAY

The protein-lipid co-sedimentation assay is based on a reported protocol [1]. Lipid mixtures of 5% PIP_2 (94.5 mol% DOPC, 5 mol% PI(4,5)P_2, and 0.5 mol% PEG2000-PE), 5% PIP_3 (94.5 mol% DOPC, 5 mol% PI(3, 4,5)P_3, and 0.5 mol% PEG2000-PE) were mixed in chloroform. Lipid films were prepared by drying the lipid mixtures under a stream of nitrogen and placed under a vacuum for at least 2 h. Lipid films were hydrated and re-suspended by vortexing for 30 min in HEPES buffer (pH 7.4, 50 mM HEPES, and 150 mM KCl) at a final lipid concentration of 1 mM. The obtained PIP_2- and PIP_3-liposomes were used within 2 h.

Protein aggregates were removed by centrifugation (436000 rcf, 30 min) at 4 °C. Protein concentrations were determined with a UV-Vis spectrometer. Combinations of kindlin/talin with PIP_2-/PIP_3-liposomes and kindlin/talin alone (negative controls) were mixed in HEPES buffer with a final concentration of 1 μM of protein, 0.5 mM of liposome, and 1 mM DTT in a volume of 50 μL. The solutions were incubated for 30 min at room temperature.

After 30-min incubation, the solutions were ultra-centrifuged at 436000 × g for 30 min at 4 °C. The supernatant was removed, and the pellet was re-suspended in 50 μL of HEPES buffer. Samples were analyzed with SDS-PAGE. The protein-bound fraction was estimated from the measured intensities from the gel images:

$$\text{Protein-bound fraction} = \frac{\text{Intensity}_{\text{Pellet}}}{\text{Intensity}_{\text{Pellet}} + \text{Intensity}_{\text{Supernatant}}}$$

PROTEIN LABELING

Atto 565-NHS-ester (Sigma-Aldrich) was dissolved in DMSO (Sigma-Aldrich) at the concentration of 5 μg/μL. Kindlin was re-buffered to phosphate-buffered saline using Zeba^{TM} Spin Desalting Columns (7K, 0.5 mL; Thermo Scientific) and mixed with the Atto 565-NHS-ester solution in a mole ratio of 1:2.5. The mixture was kept for 2 h in room temperature. After the 2-h reaction, the mixture was re-buffered to tris-buffered saline to quench the reaction and remove the excess dye. The labeled proteins were stored at -80 °C.

CO-LOCALIZATION EXPERIMENT

The sequential assembly of 1 μM kindlin and 1 μM talin followed by 200 nM paxillin and 100 nM FAK on β1-bound 5% PIP_2, 5% PIP_3, and 2.5% PIP_2+2.5% PIP_2 membranes were performed as described in the manuscript. 5 mol% labeled kindlin was used to visualize the condensates on the membranes (Figure S6).

* abausch@mytum.de

[1] Senju, Y., Lappalainen, P. & Zhao, H. *Liposome Co-sedimentation and Co-flotation Assays to Study LipidProtein Interactions*, 195–204 (Springer US, New York, NY, 2021).

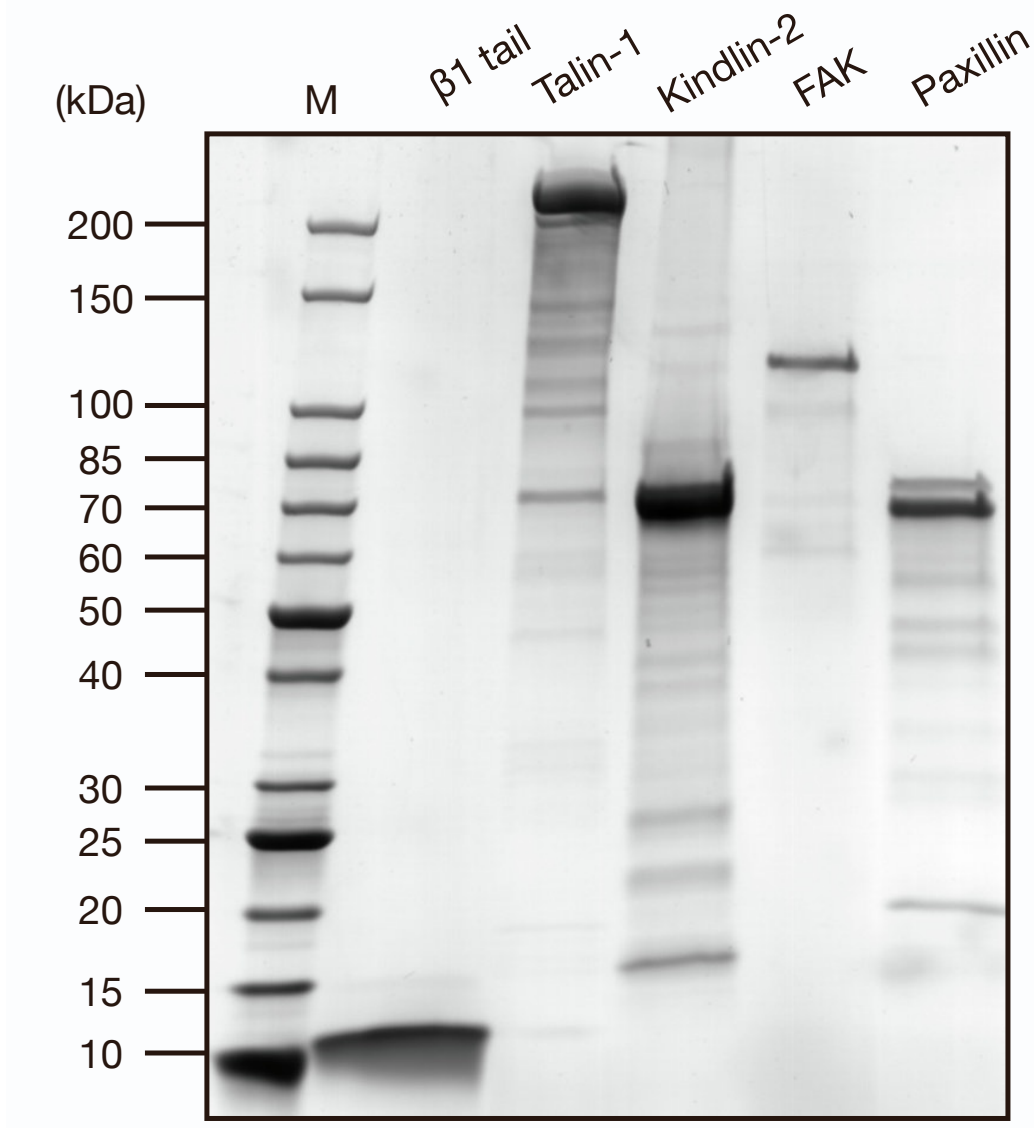

Supplementary Figure 1. **Protein characterization.** SDS-PAGE gel of purified proteins stained with Quick Coomassie.

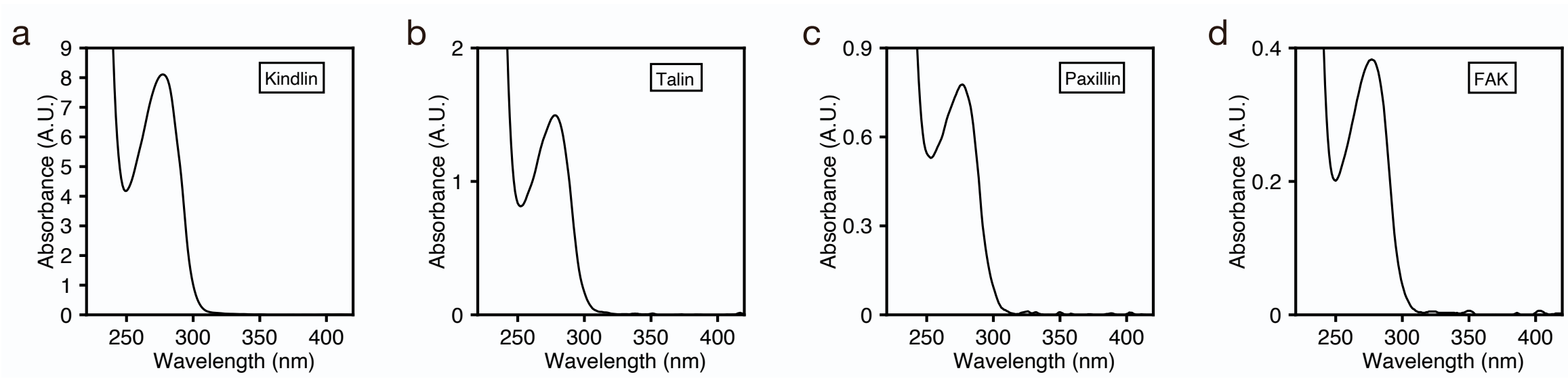

Supplementary Figure 2. **Protein characterization.** UV-Vis spectrums of kindlin (a), talin (b), paxillin (c), and FAK (d).

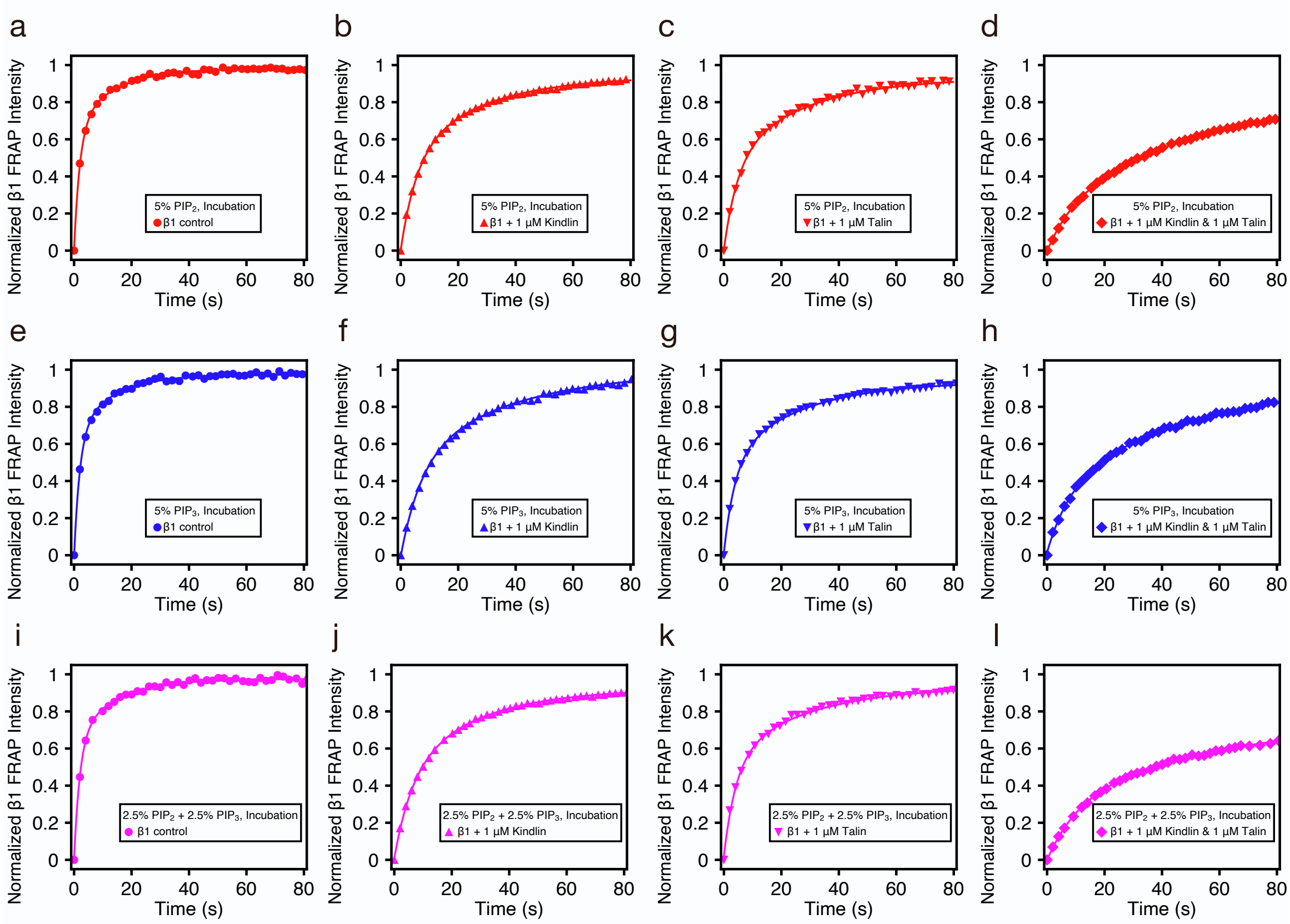

Supplementary Figure 3. **Diffusion of $\beta1$ on membranes.** Normalized FRAP measurements of $\beta1$ with 5% PIP_2 (a–d), 5% PIP_3 (e–h), and 2.5% PIP_2+2.5% PIP_2 (i–l) membranes. Solid curves show the double component exponential recovery fittings.

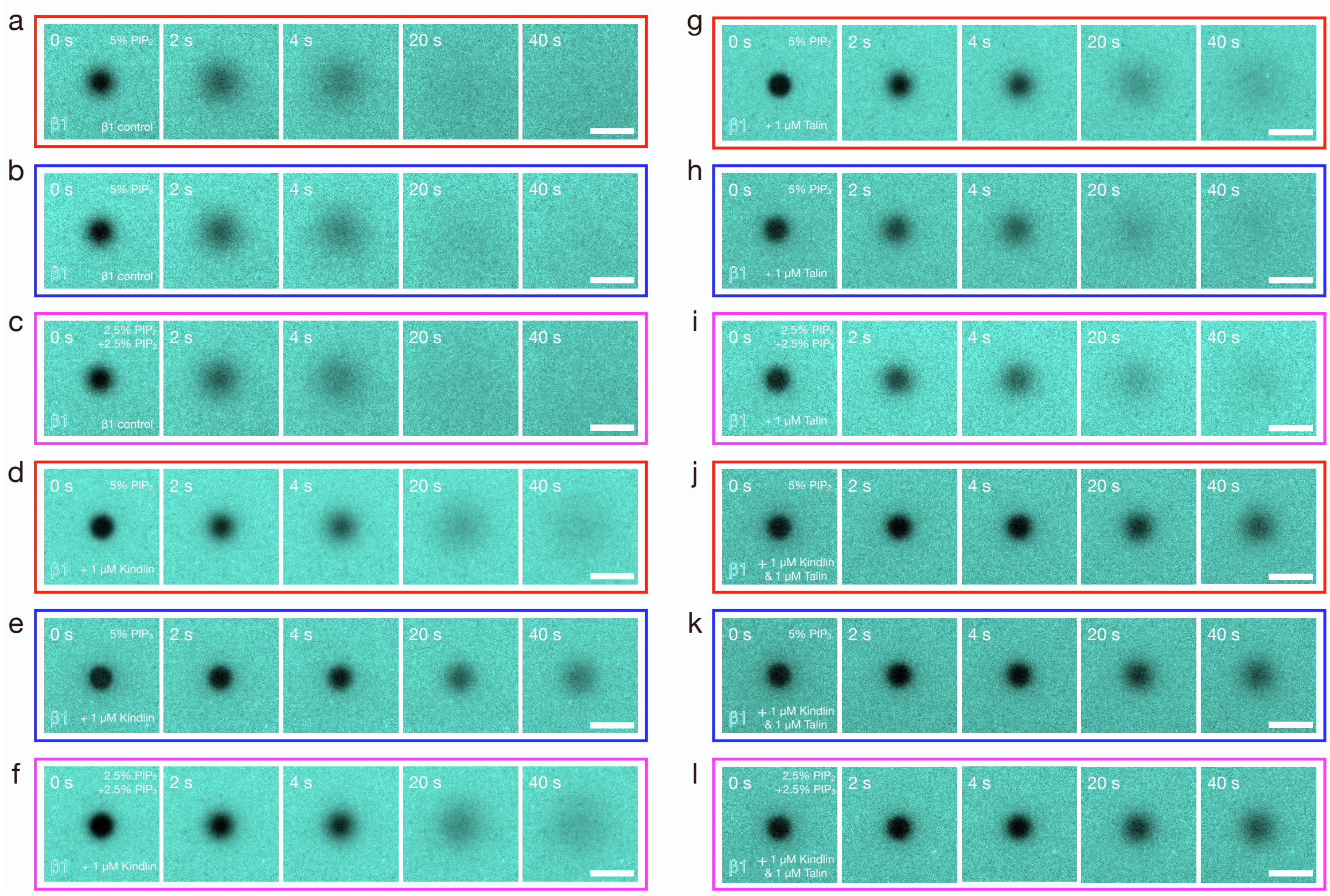

Supplementary Figure 4. **Diffusion of $\beta1$ on membranes.** FRAP images of $\beta1$ on 5% PIP_2 (a), 5% PIP_3 (b), and 2.5% PIP_2+2.5% PIP_2 (c) membranes. FRAP images of $\beta1$ after 30 min incubation with 1 μM kindlin on 5% PIP_2 (d), 5% PIP_3 (e), and 2.5% PIP_2+2.5% PIP_2 (f) membranes. FRAP images of $\beta1$ after 30 min incubation with 1 μM talin on 5% PIP_2 (g), 5% PIP_3 (h), and 2.5% PIP_2+2.5% PIP_2 (i) membranes. FRAP images of $\beta1$ after 30 min incubation with 1 μM kindlin and 1 μM talin on 5% PIP_2 (j), 5% PIP_3 (k), and 2.5% PIP_2+2.5% PIP_2 (l) membranes.

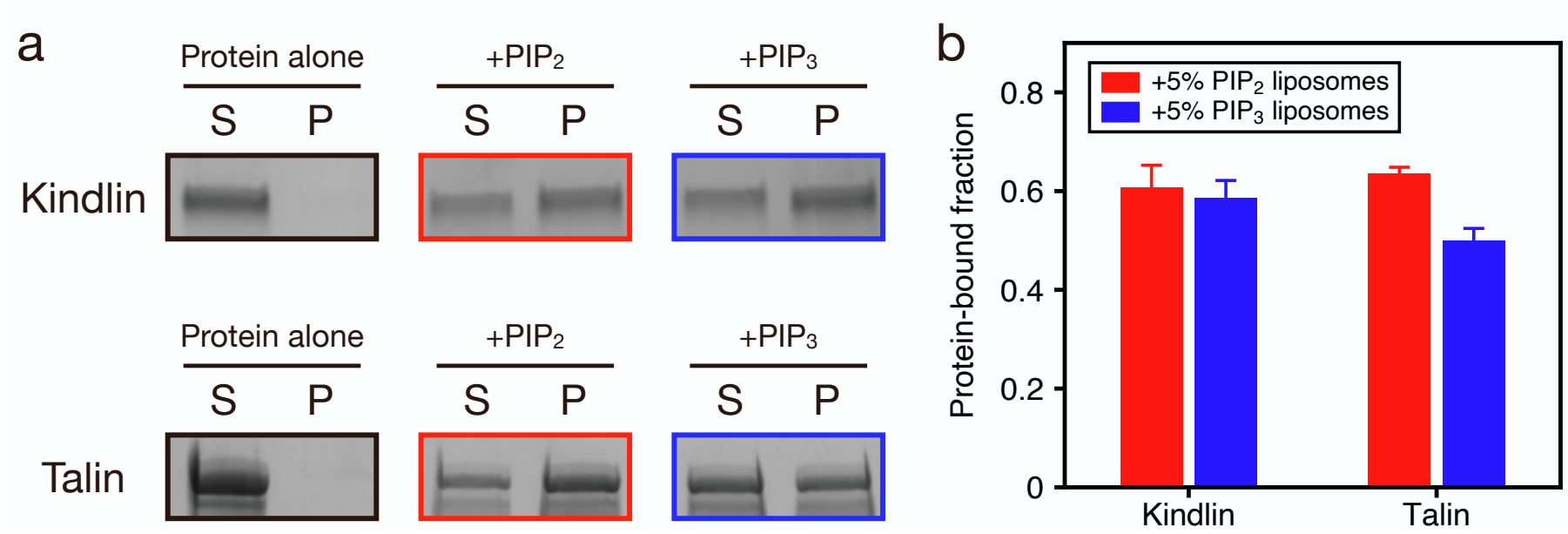

Supplementary Figure 5. **Interactions between kindlin/talin and PIP_2/PIP_3.** (a) Images of SDS-PAGE gels stained with Quick Coomassie after the PIP_2- /PIP_3-liposome co-sedimentation assay of kindlin (up) and talin (bottom). (b) Fraction of kindlin or talin bound to the PIP_2-liposomes (red) and PIP_2-liposomes (blue). Error bars represent the standard deviations from 3 measurements.

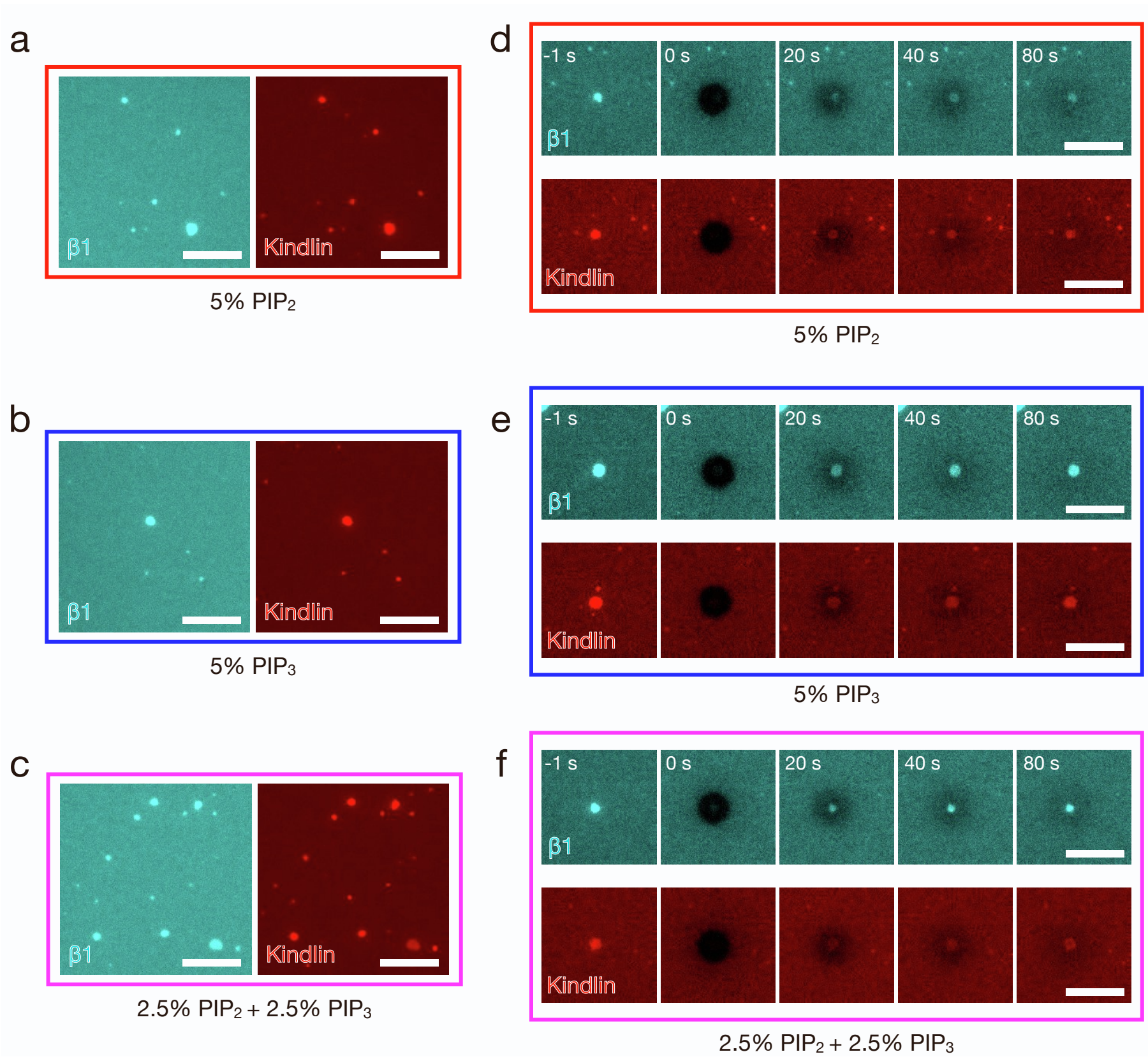

Supplementary Figure 6. **Emergence of $\beta1$ clusters.** $\beta1$ and kindlin clusters formed after sequential incubation on 5% PIP_2 (a), 5% PIP_3 (b), and 2.5% PIP_2+2.5% PIP_3 (c) membranes. FRAP images of a $\beta1$ cluster and a kindlin cluster formed on 5% PIP_2 (d), 5% PIP_3 (e), and 2.5% PIP_2+2.5% PIP_3 membranes (f). Scale bars, 10 μm.

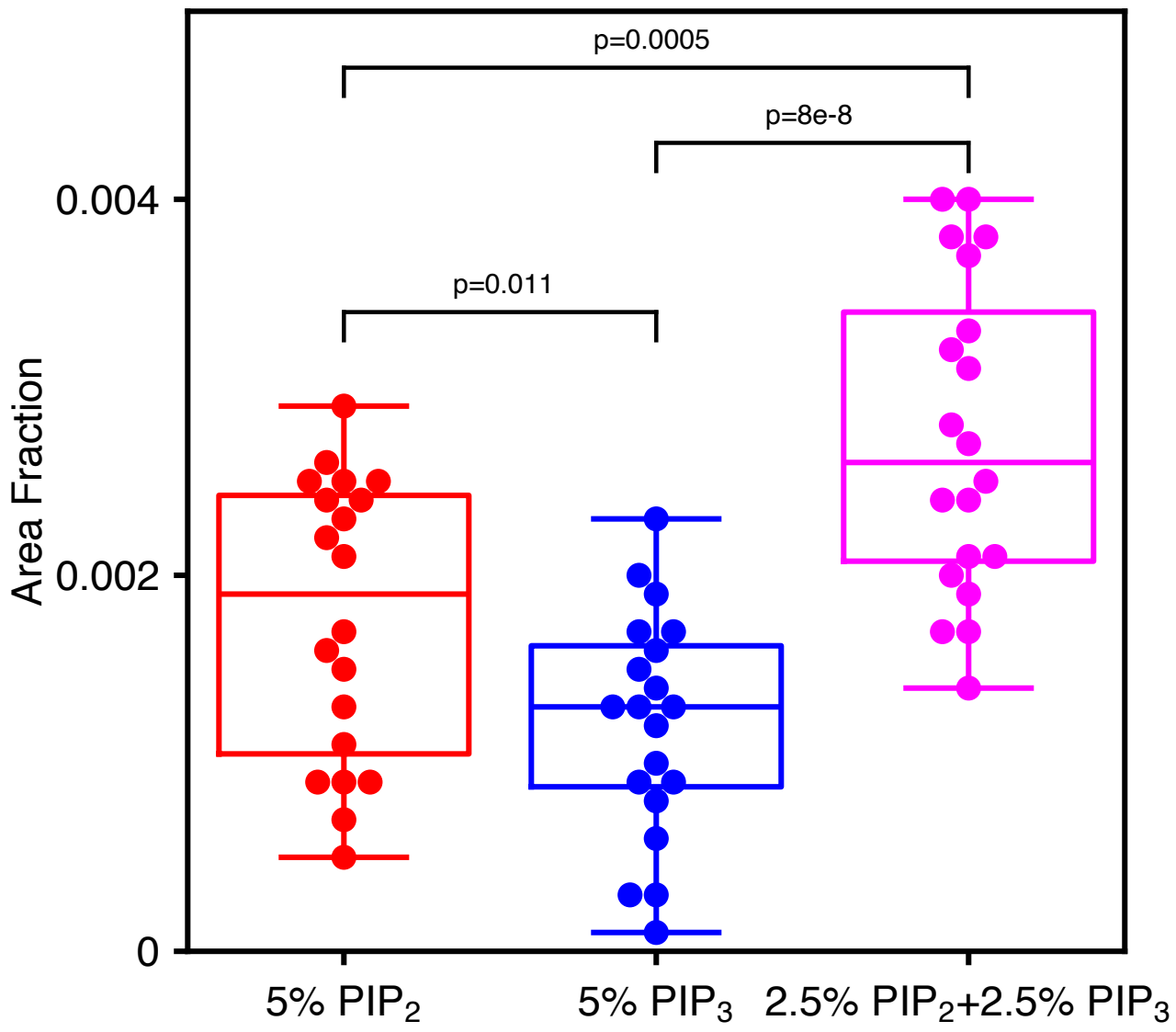

Supplementary Figure 7. **Area fraction of β1 clusters.** Area fraction of β1 clusters formed after 90-min incubation on 5% PIP_2, 5% PIP_3, and 2.5% PIP_2+2.5% PIP_2 membranes. Each condition contains 20 measurements from 4 independent experiments. Box plots indicate the median (line); 25th and 75th percentile (box); 0th and 100th percentile (whiskers). Significance tested with unpaired t-test.

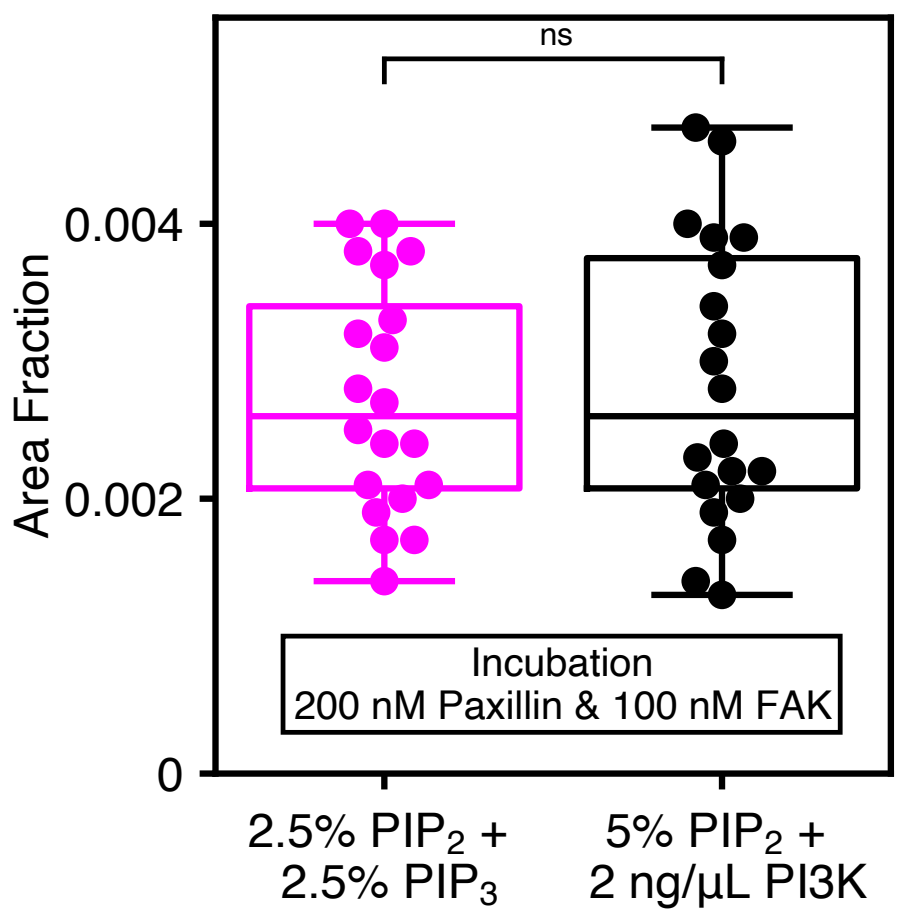

Supplementary Figure 8. **Area fraction of β1 clusters.** Area fraction of β1 clusters formed after 90-min incubation (200 nM paxillin and 100 nM FAK) on 2.5% PIP_2+2.5% PIP_2 membranes and 5% PIP_2 membranes with 2 ng/μL PI3K. Each condition contains 20 measurements from 4 independent experiments. Box plots indicate the median (line); 25th and 75th percentile (box); 0th and 100th percentile (whiskers). Significance tested with unpaired t-test.

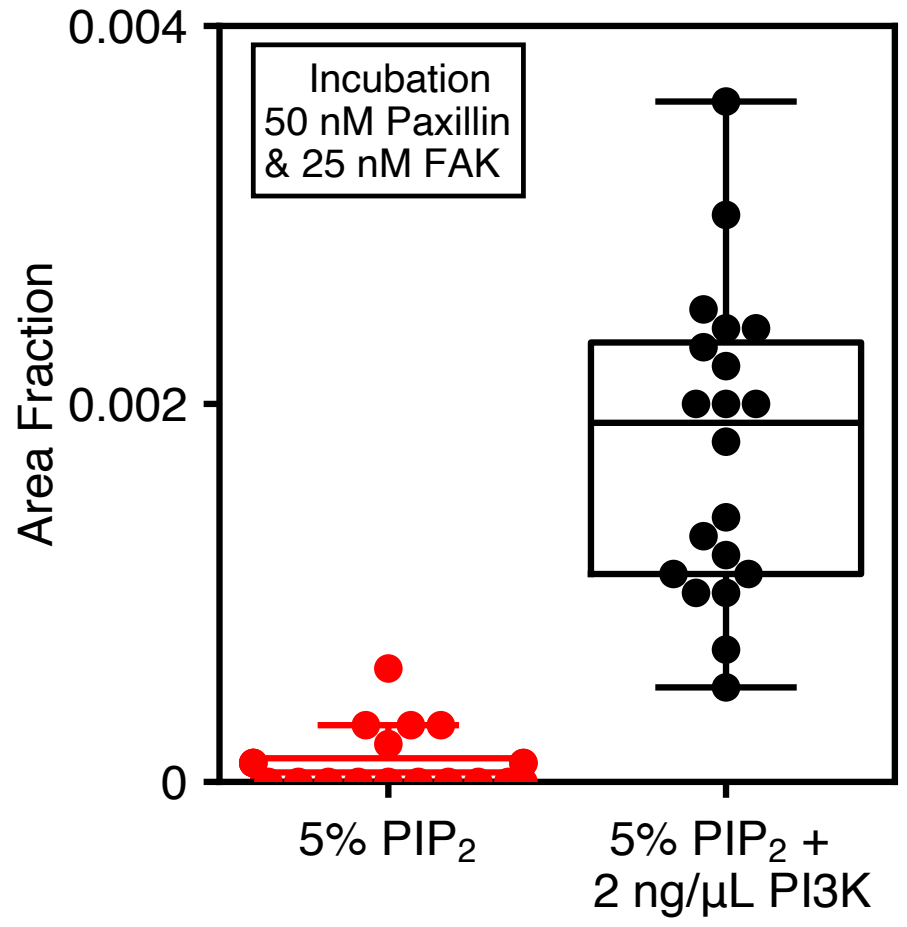

Supplementary Figure 9. **Area fraction of β1 clusters.** Area fraction of β1 clusters formed after 90-min incubation (50 nM paxillin and 25 nM FAK) on 5% PIP_2 membranes and 5% PIP_2 membranes with 2 ng/μL PI3K. Each condition contains 20 measurements from 4 independent experiments. Box plots indicate the median (line); 25th and 75th percentile (box); 0th and 100th percentile (whiskers).
