# Supplementary material for: Synergistic effect of PIP2 and PIP3 on membrane-induced phase separation of integrin complexes
Source: Biophys J. 2025 Apr 14;125(12):2936–44. doi: 10.1016/j.bpj.2025.04.011 (PMC13507320; doi:10.1016/j.bpj.2025.04.011)
Supplement: Supplementary file 2 [file mmc2.pdf]

Biophysical Journal
Article

Synergistic effect of PIP_2 and PIP_3 on membrane-induced phase separation of integrin complexes

Chiao-Peng Hsu,[1] Arsenii Hordeichyk,[1,2] Jonas Aretz,[3] Reinhard Fässler,[3] and Andreas R. Bausch[1,2,*]
[1]Heinz Nixdorf Chair for Cellular Biophysics, Center for Functional Protein Assemblies, Center for Organoid Systems, Department of Bioscience, Technical University of Munich, Technical University of Munich School of Natural Sciences, Garching, Germany; [2]Max Planck School Matter to Life, Heidelberg, Germany; and [3]Department of Molecular Medicine, Max Planck Institute of Biochemistry, Martinsried, Germany

SUMMARY The assembly of integrin adhesion complexes at the inner leaflet of the plasma membrane regulates cell adhesion to the extracellular matrix. The multivalent protein interactions within the complexes and with the cell membrane display characteristics of membrane-associated biomolecular condensates driven by liquid-liquid phase separation. The composition of lipids and the distribution of the cell membrane are crucial for forming integrin adhesion complexes. Here, we report that phosphatidylinositol 4,5-bisphosphate (PIP_2) and phosphatidylinositol (3,4,5)-trisphosphate (PIP_3) in the model membrane synergistically regulate the formation of membrane-induced integrin adhesion condensates, which consist of $\beta 1$ tails, kindlin, talin, paxillin, and focal adhesion kinase. We show that the preferential bindings of kindlin to PIP_3 and talin to PIP_2 enhance their recruitment to the membrane, which in turn increases the probability of membrane-associated phase separation. Our results indicate that modulating the intricate balance of membrane composition is a strategy to localize integrin adhesion complexes and optimize their density on lipid membranes.

SIGNIFICANCE Integrin adhesion complexes located at the inner leaflet of the plasma membrane facilitate cell adhesion to extracellular matrix proteins and activate various signaling pathways. The composition and distribution of the lipid membrane affect the formation of integrin adhesion complexes, which are driven by liquid-liquid phase separation. We show that phosphoinositides PIP_2 and PIP_3 in the model membrane synergistically govern the formation of membrane-induced integrin adhesion condensates, which consist of $\beta 1$ tails, kindlin, talin, paxillin, and focal adhesion kinase. Our findings suggest that tuning the membrane composition can effectively localize integrin adhesion complexes and enhance their density on lipid membranes.

INTRODUCTION

Integrin adhesion complexes are essential for cells to sense and respond to the biophysical and biochemical properties of the extracellular matrix, influencing cellular behaviors such as spreading, migration, polarity, differentiation, and proliferation (1,2). Integrins are α-β heterodimers composed of large ectodomains that facilitate ligand binding, single-span transmembrane helices, and short cytoplasmic tails that indirectly link with the contractile actomyosin cytoskeleton (3,4). Upon activation, integrins cluster and form nascent adhesions with early adaptor proteins at the leading edge of migrating cells, whereas the linkage to actin cytoskeleton is critical for the stabilization and maturation of nascent adhesions into focal adhesions (2,5,6). Kindlin, talin, paxillin, and focal adhesion kinase (FAK) are involved in the formation and regulation of nascent adhesions as kindlin glides along the plasma membrane and other proteins diffuse between the cytoplasm and the integrin adhesion complexes (5,7–9). The multivalent protein interactions and protein dynamics in the integrin adhesion complexes display characteristics of membrane-associated biomolecular condensates driven by liquid-liquid phase separation (10). In contrast to biomolecular condensates found in the cytoplasm and the nucleus, the spatiotemporal regulation of integrin adhesion complexes is influenced by the composition and organization of the plasma membrane (11–14). Moreover, cells can benefit from local protein enrichment, which facilitates condensate formation and function in specific locations at membranes. In vitro reconstitutions have shown that phase-separated condensates can emerge at

Submitted December 10, 2024, and accepted for publication April 10, 2025.
*Correspondence: abausch@mytum.de
Editor: Alexandra Zidovska
https://doi.org/10.1016/j.bpj.2025.04.011
© 2025 The Author(s). Published by Elsevier Inc. on behalf of Biophysical Society.
This is an open access article under the CC BY license (http://creativecommons.org/licenses/by/4.0/).

lower concentration thresholds and display slower dynamics when associated with membranes than in bulk solutions (15–17).

The composition of the cell membrane, including the types and distribution of lipids and the presence of specific lipid microdomains, is crucial for the formation and regulation of integrin adhesion complexes (12,14,18,19). In particular, the presence of specific phosphoinositides, such as phosphatidylinositol 4,5-bisphosphate (PIP_2) and phosphatidylinositol (3,4,5)-trisphosphate (PIP_3), modulate membrane dynamics and integrin function (20,21). The key adaptor proteins that bind to integrin directly, kindlin and talin, also bind to these phosphoinositides (22). Kindlin binds to PIP_2 and PIP_3 via its pleckstrin homology (PH) domain and shows a stronger preference for binding to PIP_3 than to PIP_2 (23,24). Talin binds to PIP_2 via its protein 4.1, ezrin, radixin, moesin domain, which induces a conformational change in talin to expose the integrin-binding site (25). PIP_2 is typically present in the plasma membrane at a concentration of 1%–3% of the total lipid content, whereas PIP_3 is present at much lower levels, constituting 0.01%–0.05% of the total lipid content (20,26). Although they comprise only a small fraction of the cell membrane, the balance between PIP_2 and PIP_3 in cells is dynamically regulated by a balance of kinase and phosphatase activities to ensure proper cellular function (27). PIP_2 has been shown to recruit kindlin and talin on the membrane and promote membrane-localized phase separation of nascent adhesions (17). However, the concentration of PIP_3 can increase at the leading edge of migrating cells due to the activation of phosphoinositide 3-kinase (PI3K) (20,28). How the shift of PIP_2/PIP_3 equilibrium influences the membrane-localized phase separation requires further exploration. The colocalization of PIP_3 enrichment and formation of nascent adhesions at the leading edge of migrating cells prompted us to assess the role of PIP_2 and PIP_3 balance in facilitating the phase separation of nascent adhesions.

Here, we demonstrate that kindlin has a preference for binding to PIP_3, whereas talin has a preference for binding to PIP_2. These preferences result in enhanced recruitment of kindlin and talin to the membrane, forming complexes with $\beta 1$ tails. We show that the formation of membrane-induced nascent integrin adhesion condensates, composed of $\beta 1$ tails, kindlin, talin, paxillin, and FAK, can be tuned by the presence of PIP_2 and PIP_3 in the membrane. Our results indicate that tuning the coexistence of PIP_2 and PIP_3 synergistically enhances the probability of enabling specific membrane-associated phase separation.

MATERIALS AND METHODS

Materials and reagents

Isopropyl-β-D-thiogalactopyranoside, tris, tris(2-carboxyethyl)phosphine (TCEP), hydrogen peroxide (H_2O_2), sulfuric acid (H_2SO_4), imidazole, sodium hydroxide (NaOH), 4-(2-hydroxyethyl)-1-piperazineethanesulfonic acid (HEPES), potassium chloride (KCl), zinc chloride ($ZnCl_2$), sodium azide (NaN_3), citric acid, ethylenediaminetetraacetic acid (EDTA), Atto 647 NHS-ester, dithiothreitol, glucose, pyranose-oxidase, catalase, ATP, and PI3 kinase (p120γ) were purchased from Sigma-Aldrich (St. Louis, Missouri). 1,2-dioleoyl-sn-glycero-3-phosphocholine (DOPC), 1,2-dioleoyl-sn-glycero-3-[(N-(5-amino-1-carboxypentyl)iminodiacetic acid)succinyl] (nickel salt) (DGS-NTA-Ni), 1,2-dioleoyl-sn-glycero-3-phosphoethanolamine-N-[methoxy(polyethylene glycol)-2000] (ammonium salt) (PEG2000-PE), 1,2-dioleoyl-sn-glycero-3-phospho-(1′-myo-inositol-4′,5′-bisphosphate) (ammonium salt) ($PI(4,5)P_2$), and 1,2-dioleoyl-sn-glycero-3-phospho-(1′-myo-inositol-3′,4′,5′-trisphosphate) (ammonium salt) ($PI(3,4,5)P_3$) were purchased from Avanti Polar Lipids (Alabaster, Alabama). 1,2-dihexadecanoyl-sn-glycerin-3-phosphoethanolamin (Oregon Green DHPE) was purchased from Thermo Fisher Scientific (Waltham, Massachusetts).

Buffers

HEPES buffer (pH 7.4) contains 50 mM HEPES, 1 mM dithiothreitol, 2.5 μM $ZnCl_2$, and 150 mM KCl. The buffer contains 2.5 μM $ZnCl_2$ to ensure the binding of kindlin and paxillin (9,29,30). Citric buffer (pH 4.85) contains 20 mM citric acid, 50 mM KCl, 0.1 mM EDTA, and 0.1 mM NaN_3.

Protein expression and purification

Atto643-labeled his$_6$-$\beta 1$ integrin cytoplasmic tail peptides were synthesized and purified by the Max Planck Institute of Biochemistry (MPIB) Core Facility. The correct peptide sequence was controlled by high-resolution intact mass spectrometry.

Kindlin-2 and paxillin were expressed and purified as described earlier (29). Briefly, an N-terminal his-sumo tag was added to express the proteins soluble in *E. coli* (DE3) Rosetta 2. After cell lysis, the proteins were captured with a Ni-NTA-immobilized metal ion affinity chromatography (IMAC); the sumo tag was removed by SenP2 digest and subsequent pull-down with Ni-NTA beads followed by a final size-exclusion chromatography.

His-tagged talin-1 was expressed and purified as described earlier (31) with minor changes. The protein was expressed in *E. coli* (DE3) Rosetta 2 upon induction with 0.2 mM isopropyl-β-D-thiogalactopyranoside overnight at 18°C. The cells were harvested by centrifugation and lysed by sonication in IMAC running buffer (25 mM Tris [pH 7.8], 500 mM NaCl, and 1 mM TCEP) supplemented with 2 mM $MgCl_2$, 40μ L DNaseI (obtained from the MPIB Core Facility), and a spatula tip of lysozyme. After clearing the lysate, the sample was loaded on a 5 mL HisTrap HP Ni-NTA IMAC column (17524801; Cytiva, Marlborough, Massachusetts), washed with IMAC running buffer, and eluted with a stepwise gradient with IMAC elution buffer (25 mM Tris [pH 7.8], 500 mM NaCl, 1 mM TCEP, and 500 mM imidazole). Elution fractions were pooled, concentrated, and further purified with a Superose 6 Increase 10/300 GL size-exclusion chromatograph (29091596, Cytiva) using 20 mM Tris (pH 7.8), 200 mM NaCl, and 1 mM TCEP as running buffer.

Murine FAK carrying an N-terminal his-sumo tag was cloned using Gateway cloning into PB-T-Rfa to establish a stable FAK-expressing HEK293T cell line (32). The cells were cultured in FreeStyle 293 Expression Medium (12338018, Gibco, Waltham, Massachusetts) until reaching a cell density of 10^6 cells per mL before inducing protein expression by doxycycline addition (1 μg/mL) for 3 days. Cells were collected by centrifugation, resuspended in IMAC running buffer (25 mM Tris [pH 7.5], 500 mM NaCl, 10% glycerol, and 1 mM TCEP) supplemented with a cOmplete, EDTA-free protease inhibitor tablet and 40 μL benzonase (obtained from the MPIB Core Facility) and lysed by douncing on ice. Centrifugation-cleared lysate (60 min, 58,000*g*, 4°C) was sterile filtered, applied to Ni-NTA IMAC column (HisTrap SP, 5 mL, Cytiva), washed with IMAC running buffer, and eluted with a stepwise gradient with IMAC elution

buffer (25 mM Tris [pH 7.5], 500 mM imidazole, 500 mM NaCl, 10% glycerol, and 1 mM TCEP). The protein was diafiltered using a 30 kDa cutoff Amicon Ultra 15 (UFC903024; Merck Millipore, Livingston, Scotland) filter against IMAC running buffer, and the his-sumo-tag was cleaved using sumo protease (obtained from the MPIB Core Facility) overnight at 4°C. The cleaved protein was further purified with size-exclusion chromatography to remove any protein aggregates using a Superdex 200 Increase 10/300 GL column (28-9909-44; Cytiva) in phosphate-buffered saline supplemented with additional 150 mM NaCl and 1 mM TCEP.

All protein preparations were controlled for their integrity and purity using SDS-PAGE, high-resolution intact mass spectrometry, UV-visible spectrum, and dynamic light scattering (Figs. S1 and S2).

Small unilamellar vesicle production

Lipid mixtures of 5% PIP_2 (89.3 mol % DOPC, 5 mol % DGS-NTA-Ni, 5 mol % PI(4,5)P_2, 0.5 mol % PEG2000-PE, and 0.2 mol % Oregon Green DHPE), 5% PIP_3 (89.3 mol % DOPC, 5 mol % DGS-NTA-Ni, 5 mol % PI (3,4,5)P_3, 0.5 mol % PEG2000-PE, and 0.2 mol % Oregon Green DHPE), and 2.5% PIP_2 + 2.5% PIP_3 (89.3 mol % DOPC, 5 mol % DGS-NTA-Ni, 2.5 mol % PI(4,5)P_2, 2.5 mol % PI(3,4,5)P_3, 0.5 mol % PEG2000-PE, and 0.2 mol % Oregon Green DHPE) were mixed in chloroform. Lipid films were prepared by drying the lipid mixtures under a stream of nitrogen and placed under a vacuum for at least 2 h. Lipid films were hydrated and resuspended by sonication for 30 min in citric buffer at a final lipid concentration of 0.5 mM. Small unilamellar vesicles (SUVs) were then created by extruding lipid suspensions 20 times through 100-nm-pore membrane filters (Whatman) using a mini-extruder (Avanti Polar Lipids). SUVs were stored at 4°C and used within 72 h.

Flow chamber preparation

Flow chambers (≈40 μL) that consist of coverslips (22 × 22 mm; Karlsruhe, Germany, Carl Roth) fixed to microscope slides (25 × 75 mm; Carl Roth) by three-layer parafilm were used for the assays. The coverslips were sonicated for 30 min in 3 M NaOH and rinsed with MilliQ H_2O before being cleaned in piranha solution (2:1, H_2SO_4/H_2O_2) for 10 min to render the surface hydrophilic. The piranha-cleaned coverslips were then rinsed and stored in MilliQ H_2O. The microscope slides were sonicated for 30 min in 2 wt % Hellmanex aqueous solution (Hellma, Plainview, New York) and rinsed with MilliQ H_2O before being stored in ethanol. The coverslips and microscope slides were used within 72 h after cleaning.

Model membrane system reconstitution

Supported lipid bilayers (SLBs) were prepared in flow chambers. SUVs were added to chambers at a final lipid concentration of 0.167 mM and allowed to rupture and form an SLB on the glass surface for 20 min at room temperature. Afterward, the SLBs were first washed with 0.8 mL citric buffer (33) and then 0.8 mL HEPES buffer to remove excess liposomes. SLBs were incubated with 1 μM his_6-tagged integrin β1 tails in HEPES buffer for 20 min and then washed with 0.8 mL HEPES buffer. The β1-bound SLBs were incubated with different protein mixtures. Assays with p120γ contain 2 mM ATP to ensure kinase activity. The protein mixtures contain 8 U/mL pyranose-oxidase. Depending on the assays, the 7 kU/mL catalase and 36 mM glucose function as an oxygen-scavenging system to prevent protein denature and photobleaching during fluorescence imaging.

Imaging and data acquisition

A Leica DMi8 microscope with an HC PL APO 100×/1.47 oil immersion objective was used to perform the fluorescence imaging using an ORCA-Flash 4.0 CMOS camera (C13440-20CU; Hamamatsu, Shizuoka, Japan). A Leica Infinity Scanner unit was used to perform fluorescence recovery after photobleaching (FRAP) experiments. Fiji/ImageJ was used to analyze the density of β1 clusters (34).

FRAP experiments

A circle with a diameter of 5 μm was bleached with a 638 nm laser, and fluorescence images were acquired for 80 s. A region of the SLB outside the circle was used for background subtraction. Fluorescence intensity values within bleached regions were exported using LAS X software. Data were normalized to prebleach levels and fitted to a double-component exponential recovery function, $F(t) = y_0 + A_{fast} \cdot e^{-t/\tau_{fast}} + A_{slow} \cdot e^{-t/\tau_{slow}}$, where $F(t)$ is the relative fluorescence intensity over time, $\frac{-(A_{fast}+A_{slow})}{1-(y_0+A_{fast}+A_{slow})}$ is the mobile fraction, τ_{fast} is the fast recovery time constant, and τ_{slow} is the slow recovery time constant. $t_{1/2\_fast} = \tau_{fast} \cdot ln\,2$ and $t_{1/2\_slow} = \tau_{slow} \cdot ln\,2$ are the fast and slow half-recovery times, respectively. Fitting was performed using Python3.

RESULTS

PIP_2/PIP_3-dependent slowdown of β1 diffusion in the presence of kindlin and talin

To study how phosphoinositides affect the binding of β1 tails to kindlin and talin on membranes, we reconstitute 5 mol % PIP_2, 5 mol % PIP_3, and 2.5 mol % PIP_2 + 2.5 mol % PIP_3 SLB membranes with attached β1 tails (materials and methods; Fig. 1 *A*). The diffusion of uniformly distributed β1 tails on the model membranes reflects the interaction between β1 tails, kindlin, talin, and the membrane (17,35). The diffusion of tethered β1 is correlated with its hydrodynamic radius on the membrane. The binding of β1 to proteins or lipids can result in the formation of transient and stable complexes on the membrane, which possess a larger hydrodynamic radius than a tethered β1. Thus, the measurement of β1 diffusion indicates the interactions with other components on the membrane. The diffusion of β1 tails is determined by FRAP measurements, enabling the calculation of the half-recovery time. A longer half-recovery time corresponds to slower diffusion of the β1 tails on the model membranes (materials and methods; Figs. S3 and S4). The diffusion of uniformly distributed β1 tails on the membrane depends on the incubated kindlin and talin as well as PIP_2 and PIP_3 (Fig. 1 *B*). Without kindlin and talin, β1 tail diffusion remains unaffected by PIP_2 and PIP_3, excluding a potential interaction of the β1 tail with the phosphoinositides. Although the incubation of 1 μM kindlin or 1 μM talin reduces the diffusion of β1 tails on the lipid membranes, the reduction of β1diffusibility varies within the three model membranes (Fig. 1 *B*).

The slowdown of β1 tail diffusion in the presence of 1 μM kindlin is enhanced on 5% PIP_3 membranes compared to 5% PIP_2 membranes. However, the effect is reversed in the presence of 1 μM talin (Fig. 1 *B*). The diffusion of β1 tails is 2.6 and 3.3 times slower when incubated with 1 μM kindlin on 5% PIP_2 and 5% PIP_3 membranes, respectively, compared to β1 tails alone. Conversely, when

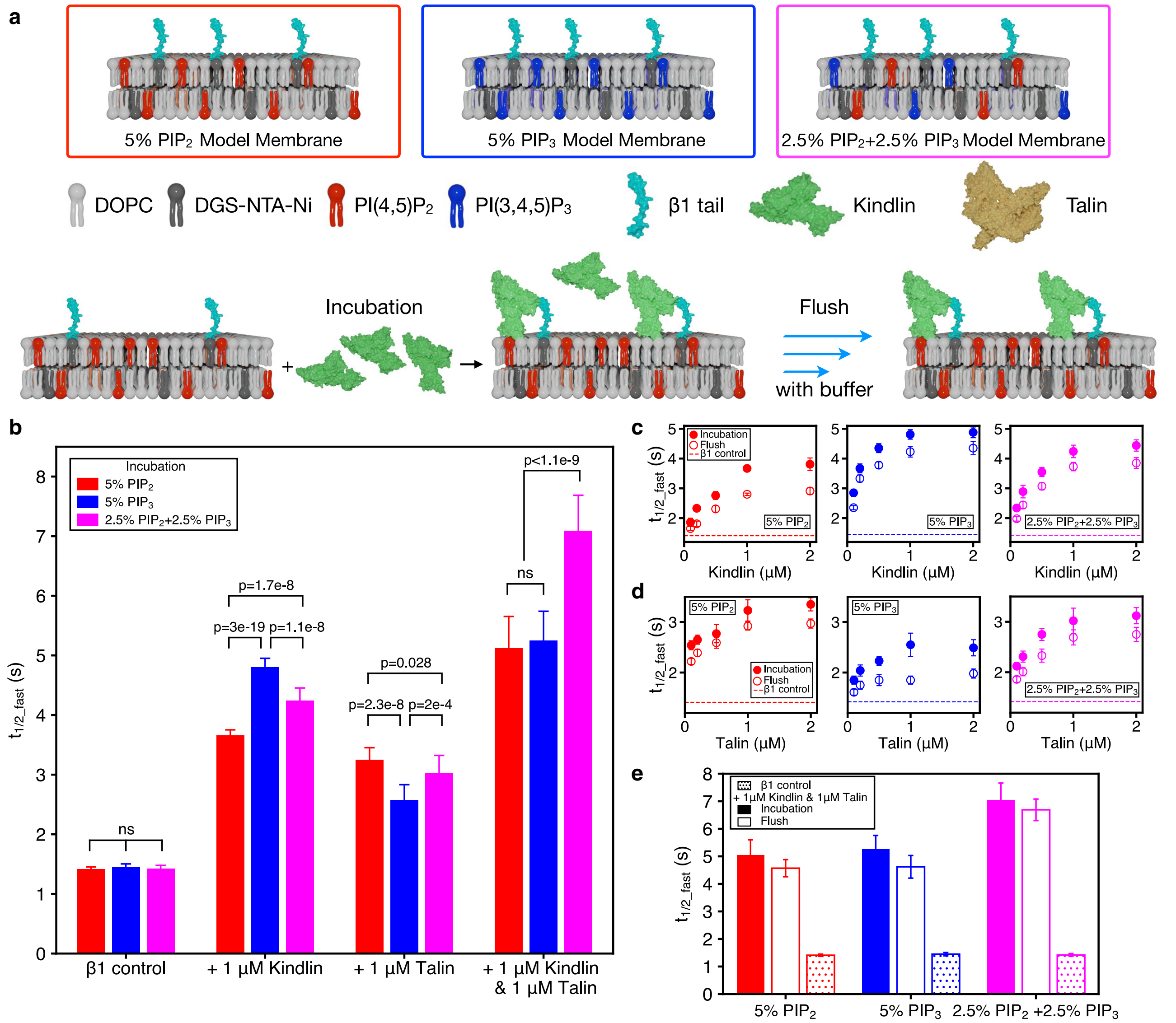

FIGURE 1 Diffusion of $\beta 1$ integrin tails on PIP_2/PIP_3 membranes. (*a*) Schematic of the reconstituted model membranes containing 5% PIP_2, 5% PIP_3, and 2.5% PIP_2 + 2.5% PIP_2, as well as the incubation and flush experiment steps with proteins. (*b*) Diffusion of $\beta 1$ ($t_{1/2\_fast}$) after 30-min incubation with kindlin/talin on 5% PIP_2, 5% PIP_3, and 2.5% PIP_2 + 2.5% PIP_2 membranes. Significance was tested with an unpaired *t*-test. (*c* and *d*) Diffusion of $\beta 1$as a function of kindlin (*c*) or talin (*d*) concentration after 30-min incubation and after flush on 5% PIP_2 (*left*), 5% PIP_3 (*middle*), and 2.5% PIP_2 + 2.5% PIP_2 (*right*) membranes. (*e*) Diffusion of $\beta 1$after 30-min incubation with 1 μM kindlin and 1 μM talin and after flush on 5% PIP_2, 5% PIP_3, and 2.5% PIP_2 + 2.5% PIP_2 membranes. Error bars in (*b*)–(*e*) represent the standard deviations of 15 measurements from 3 independent experiments.

incubated with 1 μM talin, the diffusion of $\beta 1$ tails is 2.3 and 1.8 times slower on 5% PIP_2 and 5% PIP_3 membranes, respectively, compared to $\beta 1$ tails alone. The PIP_2/PIP_3-dependent slowdown of $\beta 1$ tail diffusion on the membrane is the consequence of kindlin and talin binding to the phosphoinositides in lipid membranes via their PH and protein 4.1, ezrin, radixin, moesin domains, respectively (22–25). Although it has been identified that kindlin prefers to bind PIP_3 (23,24) and talin binds to PIP_2 (22,25), we perform co-sedimentation assays to confirm the binding between kindlin/talin and PIP_2/PIP_3 lipids used in this study (Fig. S5). In addition, these preferential interactions between kindlin, talin, and phosphoinositides have been observed with reconstituted giant unilamellar vesicles (36).

On 2.5% PIP_2 + 2.5% PIP_3 membranes, this slowdown caused by 1 μM kindlin or 1 μM talin incubation falls between the values observed for 5% PIP_2 and 5% PIP_3 membranes. Yet, the most significant reduction of $\beta 1$ tail diffusion is observed on mixed phosphoinositide membranes when incubating kindlin and talin together instead of separately. Upon incubation of 1 μM kindlin and 1 μM talin, $\beta 1$ tails diffuse 3.6 times slower on 5% PIP_2 and 5% PIP_3 membranes and 4.9 times slower on 2.5% PIP_2 + 2.5% PIP_3 membranes. The reduction in diffusibility

and mobile fraction of β1 tails on these three membranes (Fig. 1 *B*; Fig. S1) suggests that ternary β1-kindlin-talin complexes form on these membranes.

Kindlin and talin are recruited to the lipid membrane due to the presence of phosphoinositides, which effectively enhances their local concentration and binding probability to β1 tails. To further evaluate the off-rate of kindlin (Fig. 1 *C*) and talin (Fig. 1 *D*) to β1 tails on phosphoinositide membranes, we compare the diffusion of β1 tails after a 30-min incubation and then after flushing with buffer. In these experiments, 0.1–2 μM of kindlin or talin are incubated individually with the three model membranes. On all three phosphoinositide membranes, the diffusion of β1 tails after flushing decreases slightly and is slower than the diffusion of β1 tails alone. This indicates that kindlin, as well as talin alone, can already form stable complexes with β1on the 5% PIP_2, 5% PIP_3, and 2.5% PIP_2 + 2.5% PIP_3 membranes. Although kindlin shows a stronger preference for binding PIP_3 compared to PIP_2, talin has the opposite preference. On 5% PIP_3 membranes, all diffusion times of β1incubated with kindlin are longer than on 5% PIP_2 membranes. In contrast, all diffusion times of β1incubated with talin are longer on 5% PIP_2 membranes than on 5% PIP_3 membranes.

Despite the different preferences of kindlin and talin on PIP_2 and PIP_3, the 2.5% PIP_2 + 2.5% PIP_3 membranes effectively recruit these proteins (Figs. 1, *C* and *D*). The simultaneous incubation with kindlin and talin leads to larger complexes with β1 tails in the presence of phosphoinositide, as shown by the increased diffusion time after flushing (Fig. 1 *E*). These complexes lead to 3.2 times slower diffusion of β1 tails compared to the diffusion of β1 tails in the absence of kindlin or talin on 5% PIP_2 and 5% PIP_3 membranes. Synergistically, on mixed phosphoinositide membranes, the β1 tails diffuse 4.7 times slower in the same condition, indicating more β1-kindlin-talin complex formation on the membranes. The formation of β1-kindlin-talin complexes on these model membranes prompts us to explore how the phosphoinositide membrane can induce the recruitment of paxillin and FAK from the solution to the membrane to form minimal integrin adhesion complexes (17,37).

PIP_2 and PIP_3 enhance the emergence of β1 clusters on membranes

It has been shown that the sequential assembly of kindlin and talin followed by paxillin and FAK can generate surface-induced condensate formation, which leads to the clustering of β1 tails on PIP_2 membranes (17). To determine whether the condensate formation depends on different phosphoinositides in the membranes, we reconstitute the β1-kindlin-talin complexes on 5% PIP_2, 5% PIP_3, and 2.5% PIP_2 + 2.5% PIP_3 membranes via incubation and after the flushing steps (Fig. 2 *A*). After the incubation of 200 nM paxillin and 100 nM FAK at a high ionic strength of 150 mM KCl, mimicking physiological conditions, β1 tail clusters emerge spontaneously on all phosphoinositide membranes (Fig. 2 *A*). The colocalization of β1and kindlin indicates the partitioning of kindlin in these surface-induced condensates, and FRAP measurements demonstrate that β1 clusters are dynamic assemblies where β1 tails diffuse freely and exchange on the lipid membrane (Fig. S6). The emergence of β1 tail clusters suggests that the local biochemical environment of the membranes induces the phase separation of paxillin and FAK. Yet, the density and area fraction of β1 clusters depend on the phosphoinositides in the membranes (Fig. 2 *B*; Fig. S7). The β1cluster density on 2.5% PIP_2 + 2.5% PIP_3 membranes increases 1.5- and 2-fold compared to the ones on 5% PIP_2 and 5% PIP_3 membranes, respectively (Fig. 2 *B*). Our observations show that the mix

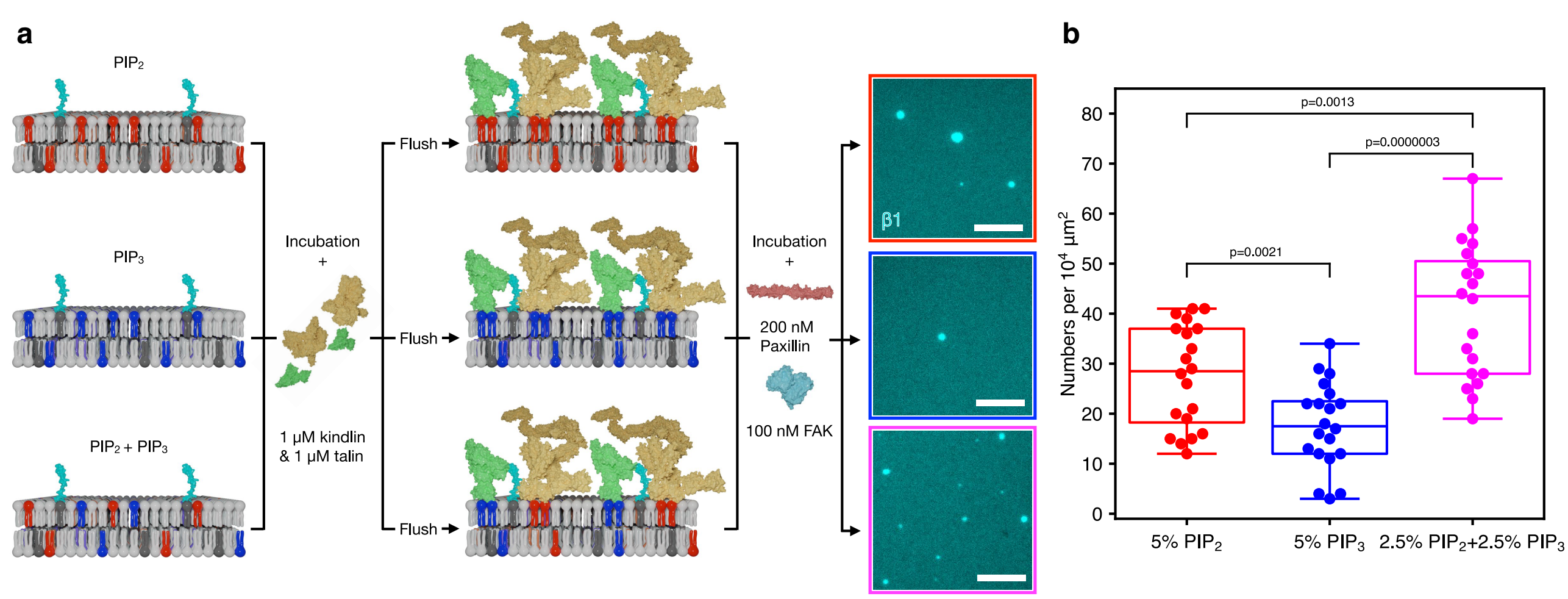

FIGURE 2 PIP_2/PIP_3 membrane synergistically enhances the emergence of β1 clusters. (*a*) Sequential incubation procedure leads to the emergence of β1 clusters after 90-min incubation on 5% PIP_2, 5% PIP_3, and 2.5% PIP_2 + 2.5% PIP_2 membranes. Scale bars, 10 μm. (*b*) The density of β1 clusters formed after 90-min incubation with the three membranes in (*a*). Each condition contains 20 measurements from 4 independent experiments. Boxplots indicate the median (*line*), 25th and 75th percentile (*box*), and 0th and 100th percentile (*whiskers*). Significance was tested with an unpaired *t*-test.

of PIP_2 and PIP_3 in the lipid membrane optimizes the local environment to increase the probability of surface-induced phase separation. Moreover, the intricate balance of PIP_2 and PIP_3 in the cell membranes motivates us to investigate how the conversion of PIP_2 and PIP_3 impacts the emergence of β1 clusters.

Balance of PIP_2 and PIP_3 regulates the emergence of β1 clusters on membranes

The balance between PIP_2 and PIP_3 in cells is tightly regulated by PI3K together with phosphatase and tensin homolog (PTEN) (27). PI3K converts PIP_2 to PIP_3 by adding a phosphate group at the 3-position, and PTEN converts PIP_3 back to PIP_2 by removing the 3-phosphate. Therefore, PIP_2 model membranes can be transformed to PIP_3 model membranes with PI3K (Fig. 3 *A*). We measure the diffusion of β1 tails with 1 μM of kindlin or talin and 0.5–10 ng/μL of PI3K after 30-min incubation on 5% PIP_2 membranes (Fig. 3 *B*). At low concentrations of PI3K (0.5 ng/μL), the diffusion of β1 tails is close to that measured on 5% PIP_2 membranes (Fig. 1 *B*). With increasing concentrations of PI3K, the diffusion of β1 tails is either slower or faster depending on the incubation with either kindlin or talin. At above 5 ng/μL of PI3K, the diffusion of β1 tails resembles that measured on 5% PIP_3 membranes (Fig. 1 *B*). The change of β1 tail diffusibility with the incubation of kindlin or talin indicates the transformation of PIP_2 membranes to PIP_3 membranes. Particularly, at 2 ng/μL of PI3K, the measured fast recovery times suggest the equal-molarity coexistence of PIP_2 and PIP_3 in the model membranes, enabled by the kinase activity. We then perform sequential assembly experiments where 2 ng/μL of PI3K is added to 1 μM kindlin and 1 μM talin on 5% PIP_2 membranes. The β1 tail clusters emerge after the addition of 200 nM paxillin and 100 nM FAK, and the density and area fraction are comparable with the ones that emerge on 2.5% PIP_2 + 2.5% PIP_3 membranes (Fig. 3 *C*; Fig. S8). Moreover, at lower concentrations of paxillin and FAK (50 nM paxillin and 25 nM FAK), barely any β1 clusters form on the 5% PIP_2 membrane, but β1 clusters can be observed on the 5% PIP_2 membrane with 2 ng/μL PI3K (Fig. 3 *D*; Fig. S9). This observation demonstrates that the addition of PI3K shifts the phase separation boundary of the membrane-bound condensate system. These results indicate that the balance of PIP_2 and PIP_3 plays a key role in promoting the formation of membrane-associated nascent integrin adhesion condensates. The presence of both PIP_2 and PIP_3 enhances the probability of surface-induced phase separation.

DISCUSSION

In the present paper, we show that the balance of PIP_2 and PIP_3 determines the density of nascent integrin clusters on lipid membranes under surface-induced phase separation conditions. The slowdown of β1 tail diffusion on the lipid membrane indicates that kindlin prefers to bind PIP_3, and talin prefers to bind PIP_2. Although the complex formation of kindlin, talin, and β1 tails (38) on PIP_2- and PIP_3-only membranes can induce the recruitment and phase separation of paxillin and FAK, the highest probability of finding nascent integrin clusters is observed on mixed phosphoinositide

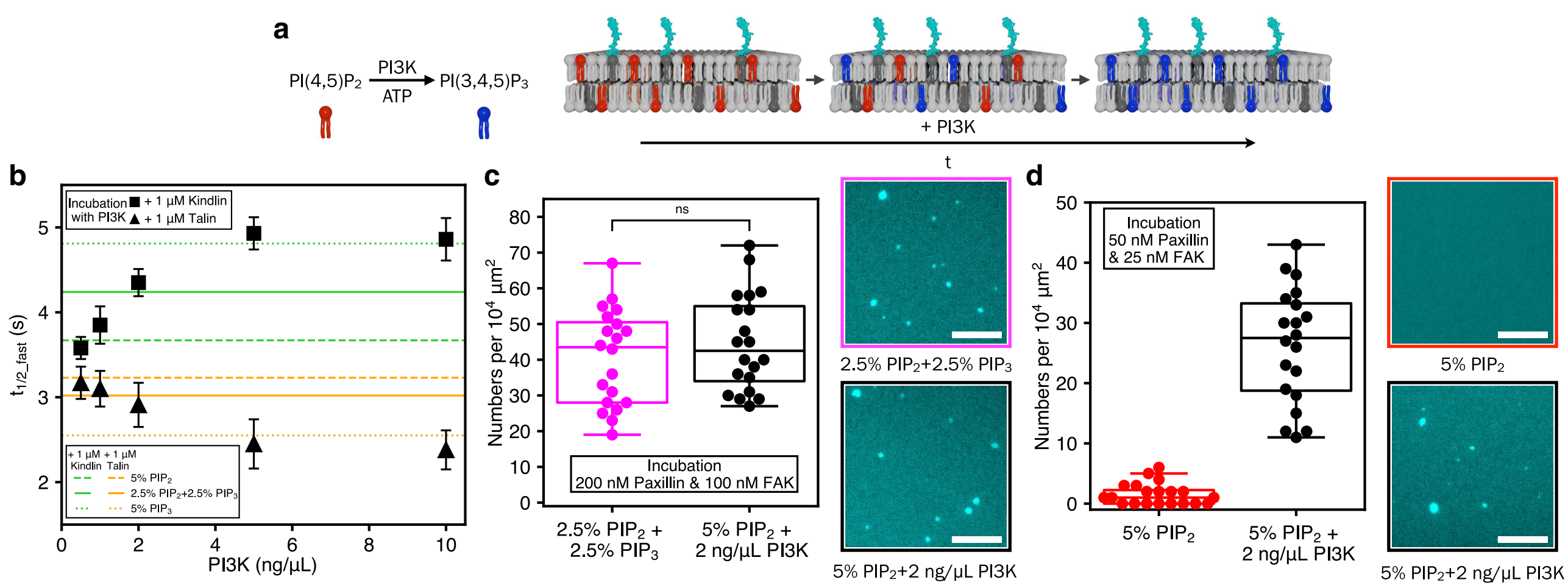

FIGURE 3 PI3 kinase modifies the membrane surfaces and regulates the emergence of β1 clusters. (*a*) Schematic of PI3 kinase catalyzes the conversion of $PI(4,5)P_2$ into $PI(3,4,5)P_3$. (*b*) Diffusion of β1as a function PI3 kinase concentration after 30-min incubation with kindlin or talin. (*c*) The density (*left*) and images (*right*) of β1 clusters formed after 90-min sequential incubation (200 nM paxillin and 100 nM FAK) on 2.5% PIP_2 + 2.5% PIP_2 membranes (same data as Fig. 2 *B*) and 5% PIP_2 membranes with 2 ng/μL PI3K. (*d*) The density (*left*) and images (*right*) of β1 clusters formed after 90-min sequential incubation (50 nM paxillin and 25 nM FAK) on 5% PIP_2 membranes and 5% PIP_2 membranes with 2 ng/μL PI3K. (*c* and *d*) Each condition contains 20 measurements from 4 independent experiments. Boxplots indicate the median (*line*), 25th and 75th percentile (*box*), and 0th and 100th percentile (*whiskers*). Significance was tested with an unpaired *t*-test. Scale bars, 10 μm.

model membranes with the same protein concentrations and assembling sequence. This synergistic observation is confirmed by shifting the PIP_2/PIP_3 equilibrium in the lipid membrane via PI3K. Thus, the balance of PIP_2 and PIP_3, regulated by PI3K and PTEN, in the lipid membrane is crucial for the effective localization and assembly of reconstituted minimal adhesion complexes (Fig. 4). Although PIP_2 alone can lower the concentration thresholds for initiating the adhesion complex via surface-induced phase separation (17), our results suggest that PIP_3 acts as an additional tuning knob to increase the probability of forming nascent adhesion sites. Unlike bulk condensates wetting the membranes (39), the surface-induced membrane-bound condensates resemble prewetting transitions on the surface (40,41). In prewetting transitions, a bulk phase below its saturation concentration is stabilized through favorable interactions with a surface, leading to the formation of a surface film. Moreover, below the saturation concentration, surface binding modulates bound and unbound molecules near the surface, resulting in various surface states and complex surface phase diagrams (42,43). With fixed protein concentrations in bulk, having two regulating knobs on the membrane, PIP_2 and PIP_3, potentially serves as a strategy to localize adhesion sites and optimize their density on lipid membranes. The ratio of PIP_2 and PIP_3 determines the levels of kindlin and talin on the membrane, which sets the kindlin/talin ratio (Fig. 4). The ratio of kindlin/talin in nascent adhesion is 2:1 and becomes 1:1 when adhesion sites start to mature (5). The imbalance of PIP_2 and PIP_3 can disrupt kindlin-talin cooperativity and lead to the disassembly of focal adhesions (38). To obtain insights on how protein condensates couple with the membranes (44), several in vitro studies have investigated the phase separation of focal adhesion proteins on SLBs. Kindlin, paxillin, and FAK have been shown to spontaneously phase separate in bulk and be bound by integrin tails to phosphoinositides-free membranes (45). Furthermore, PIP_2-containing membranes trigger the phase separation of focal adhesion proteins on the membrane surface, capturing the formation of nascent adhesions with low protein concentrations (17,46). Our model membrane reconstitutions highlight the importance of tuning the membrane compositions to form and regulate integrin adhesion complexes.

The diversity of membrane lipid composition is key to the various signaling pathways and cell functions (47). In cells, the phosphoinositide binding motifs in kindlin and talin are essential for assembling focal adhesion sites (13,48). Although the plasma membrane comprises only a small fraction of PIP_2 and PIP_3, the conversion of PIP_2 to PIP_3 via PI3K is crucial for cell signaling and migration (49). We show that the density of minimal adhesion complexes can be maximized on PI3K-modulated mixed phosphoinositides membranes. In *Dictyostelium*, the spatial restriction of PIP_3 at the leading edge is responsible for cell migration, and the lack of PI3Ks reduces the migration efficiency (50). The redistribution of PIP_3 at the cell front, using optical control, has been shown to initiate immune cell migration (51). Although PIP_3 facilitates the formation of membrane-induced nascent adhesion complexes compared to PIP_2-only membranes, its role in localizing and activating other signaling proteins involved in adhesion maturation is equally significant. PIP_3 serves as a docking site for proteins with PH domains, such as 3-phosphoinositide-dependent protein kinase-1 (PDK1) and protein kinase B (AKT). PDK1 and AKT are essential for the signaling pathways that regulate focal adhesion dynamics and cytoskeletal organization (52,53). Further reconstitutions, including the linkage to actomyosin and signaling proteins, need to be examined in future studies to identify the role of PIP_3 in developing focal adhesions. In addition to phosphoinositides, other plasma membrane compositions modulate cell adhesion dynamics, such as cholesterol and sphingolipids (54,55). Lipid domains enriched in cholesterol and sphingolipids can concentrate integrins and other focal adhesion proteins, facilitating their clustering and promoting the assembly and function of focal adhesions. How different lipid

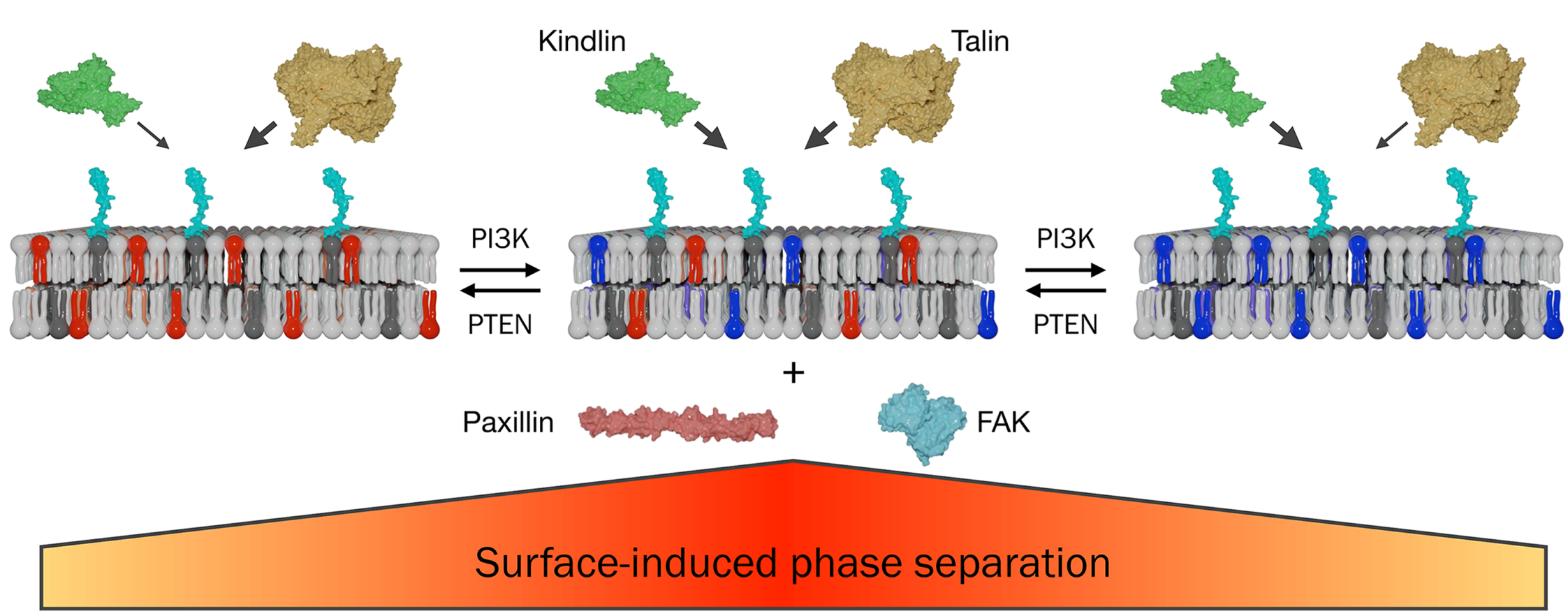

FIGURE 4 PIP_2/PIP_3 cycle sets membrane surfaces to steer the phase separation. Schematic of the balance between PIP_2 and PIP_3 optimizes the surface-induced phase separation.

membranes orchestrate to form adhesion complexes can be explored with the membrane assay presented in this study.

By influencing the localization, conformation, and activity of membrane proteins, lipids play a critical role in maintaining cellular equilibrium and responding to environmental cues (21). This study sets a framework for understanding how complex lipids synergistically modulate the formation of adhesion complexes. Understanding further protein-membrane interactions provides insights into the molecular mechanisms underlying many physiological processes and diseases governed by cell adhesions.

ACKNOWLEDGMENTS

We dedicate this work to the memory of Erich Sackmann, whose teaching, mentorship, and friendship profoundly influenced our research. His unwavering support, insightful guidance, and dedication to biophysics and science have been instrumental in shaping our research.

This paper stands as a testament to his lasting impact, and his legacy will continue to inspire and guide future endeavors.

We gratefully acknowledge financial support from the European Research Council (ERC) under the European Union's Horizon 2020 research and innovation programme (grant agreement no. 810104-PoInt). This research was conducted within the Max Planck School Matter to Life, supported by the German Federal Ministry of Education and Research (BMBF) in collaboration with the Max Planck Society.

AUTHOR CONTRIBUTIONS

C.-P.H., R.F., and A.R.B. designed the research; C.-P.H., A.H., and J.A. performed the research; C.-P.H. and A.H. analyzed the data; and C.-P.H. and A.R.B. wrote the manuscript. All authors reviewed and revised the manuscript.

DECLARATION OF INTERESTS

The authors declare no competing interests.

SUPPORTING MATERIAL

Supporting material can be found online at https://doi.org/10.1016/j.bpj.2025.04.011.

REFERENCES

1. Winograd-Katz, S. E., R. Fässler, …, K. R. Legate. 2014. The integrin adhesome: from genes and proteins to human disease. *Nat. Rev. Mol. Cell Biol.* 15:273–288.
2. Sun, Z., M. Costell, and R. Fässler. 2019. Integrin activation by talin, kindlin and mechanical forces. *Nat. Cell Biol.* 21:25–31.
3. Moser, M., K. R. Legate, …, R. Fässler. 2009. The tail of integrins, talin, and kindlins. *Science.* 324:895–899.
4. Campbell, I. D., and M. J. Humphries. 2011. Integrin structure, activation, and interactions. *Cold Spring Harb. Perspect. Biol.* 3:a004994.
5. Bachir, A. I., J. Zareno, …, A. R. Horwitz. 2014. Integrin-Associated Complexes Form Hierarchically with Variable Stoichiometry in Nascent Adhesions. *Curr. Biol.* 24:1845–1853.
6. Kumari, R., K. Ven, …, P. Lappalainen. 2024. Focal adhesions contain three specialized actin nanoscale layers. *Nat. Commun.* 15:2547.
7. Lawson, C., S. T. Lim, …, D. D. Schlaepfer. 2012. FAK promotes recruitment of talin to nascent adhesions to control cell motility. *J. Cell Biol.* 196:223–232.
8. Changede, R., X. Xu, …, M. P. Sheetz. 2015. Nascent Integrin Adhesions Form on All Matrix Rigidities after Integrin Activation. *Dev. Cell.* 35:614–621.
9. Theodosiou, M., M. Widmaier, …, R. Fässler. 2016. Kindlin-2 cooperates with talin to activate integrins and induces cell spreading by directly binding paxillin. *Elife.* 5:e10130.
10. Case, L. B., J. A. Ditlev, and M. K. Rosen. 2019. Regulation of Transmembrane Signaling by Phase Separation. *Annu. Rev. Biophys.* 48:465–494.
11. Banani, S. F., H. O. Lee, …, M. K. Rosen. 2017. Biomolecular condensates: organizers of cellular biochemistry. *Nat. Rev. Mol. Cell Biol.* 18:285–298.
12. Legate, K. R., S. Takahashi, …, R. Fässler. 2011. Integrin adhesion and force coupling are independently regulated by localized PtdIns(4,5)2 synthesis. *EMBO J.* 30:4539–4553.
13. Lu, F., L. Zhu, …, J. Qin. 2022. Mechanism of integrin activation by talin and its cooperation with kindlin. *Nat. Commun.* 13:2362.
14. Tachibana, H., K. Minoura, …, N. Kioka. 2023. The plasma membrane of focal adhesions has a high content of cholesterol and phosphatidylcholine with saturated acyl chains. *J. Cell Sci.* 136:jcs260763.
15. Ditlev, J. A. 2021. Membrane-associated phase separation: organization and function emerge from a two-dimensional milieu. *J. Mol. Cell Biol.* 13:319–324.
16. Snead, W. T., A. P. Jalihal, …, A. S. Gladfelter. 2022. Membrane surfaces regulate assembly of ribonucleoprotein condensates. *Nat. Cell Biol.* 24:461–470.
17. Hsu, C. P., J. Aretz, …, A. R. Bausch. 2023. Surface-induced phase separation of reconstituted nascent integrin clusters on lipid membranes. *Proc. Natl. Acad. Sci. USA.* 120:e2301881120.
18. Leitinger, B., and N. Hogg. 2002. The involvement of lipid rafts in the regulation of integrin function. *J. Cell Sci.* 115:963–972.
19. Goni, G. M., C. Epifano, …, D. Lietha. 2014. Phosphatidylinositol 4,5-bisphosphate triggers activation of focal adhesion kinase by inducing clustering and conformational changes. *Proc. Natl. Acad. Sci. USA.* 111:E3177–E3186.
20. Insall, R. H., and O. D. Weiner. 2001. PIP3, PIP2, and cell movement–similar messages, different meanings? *Dev. Cell.* 1:743–747.
21. Di Paolo, G., and P. De Camilli. 2006. Phosphoinositides in cell regulation and membrane dynamics. *Nature.* 443:651–657.
22. Calderwood, D. A., I. D. Campbell, and D. R. Critchley. 2013. Talins and kindlins: partners in integrin-mediated adhesion. *Nat. Rev. Mol. Cell Biol.* 14:503–517.
23. Liu, J., K. Fukuda, …, J. Qin. 2011. Structural Basis of Phosphoinositide Binding to Kindlin-2 Protein Pleckstrin Homology Domain in Regulating Integrin Activation. *J. Biol. Chem.* 286:43334–43342.
24. Liu, Y., Y. Zhu, …, R. Zhang. 2012. Crystal structure of kindlin-2 PH domain reveals a conformational transition for its membrane anchoring and regulation of integrin activation. *Protein Cell.* 3:434–440.
25. Chinthalapudi, K., E. S. Rangarajan, and T. Izard. 2018. The interaction of talin with the cell membrane is essential for integrin activation and focal adhesion formation. *Proc. Natl. Acad. Sci. USA.* 115:10339–10344.
26. McLaughlin, S., J. Wang, …, D. Murray. 2002. PIP2 and proteins: interactions, organization, and information flow. *Annu. Rev. Biophys. Biomol. Struct.* 31:151–175.
27. Czech, M. P. 2000. PIP2 and PIP3: complex roles at the cell surface. *Cell.* 100:603–606.
28. Kovacs, E. M., R. G. Ali, …, A. S. Yap. 2002. E-cadherin homophilic ligation directly signals through Rac and phosphatidylinositol 3-kinase to regulate adhesive contacts. *J. Biol. Chem.* 277:6708–6718.

29. Bottcher, R. T., M. Veelders, …, R. Fassler. 2017. Kindlin-2 recruits paxillin and Arp2/3 to promote membrane protrusions during initial cell spreading. *J. Cell Biol.* 216:3785–3798.

30. Zhu, L., H. Liu, …, J. Qin. 2019. Structural Basis of Paxillin Recruitment by Kindlin-2 in Regulating Cell Adhesion. *Structure.* 27:1686–1697.e5.

31. Sun, Z., H. Y. Tseng, …, R. Fässler. 2016. Kank2 activates talin, reduces force transduction across integrins and induces central adhesion formation. *Nat. Cell Biol.* 18:941–953.

32. Li, Z., I. P. Michael, …, J. M. Rini. 2013. Simple piggyBac transposon-based mammalian cell expression system for inducible protein production. *Proc. Natl. Acad. Sci. USA.* 110:5004–5009.

33. Braunger, J. A., C. Kramer, …, C. Steinem. 2013. Solid supported membranes doped with PIP2: influence of ionic strength and pH on bilayer formation and membrane organization. *Langmuir.* 29:14204–14213.

34. Schindelin, J., I. Arganda-Carreras, …, A. Cardona. 2012. Fiji: an open-source platform for biological-image analysis. *Nat. Methods.* 9:676–682.

35. Vinogradova, O., J. Vaynberg, …, J. Qin. 2004. Membrane-mediated structural transitions at the cytoplasmic face during integrin activation. *Proc. Natl. Acad. Sci. USA.* 101:4094–4099.

36. Pernier, J., M. Cardoso Dos Santos, …, C. Le Clainche. 2023. Talin and kindlin cooperate to control the density of integrin clusters. *J. Cell Sci.* 136:jcs260746.

37. Hoffmann, J. E., Y. Fermin, …, E. Zamir. 2014. Symmetric exchange of multi-protein building blocks between stationary focal adhesions and the cytosol. *Elife.* 3:e02257.

38. Aretz, J., M. Aziz, …, R. Fässler. 2023. Talin and kindlin use integrin tail allostery and direct binding to activate integrins. *Nat. Struct. Mol. Biol.* 30:1913–1924.

39. Mangiarotti, A., and R. Dimova. 2024. Biomolecular Condensates in Contact with Membranes. *Annu. Rev. Biophys.* 53:319–341.

40. Cahn, J. W. 1977. Critical point wetting. *J. Chem. Phys.* 66:3667–3672.

41. Nakanishi, H., and M. E. Fisher. 1982. Multicriticality of Wetting, Prewetting, and Surface Transitions. *Phys. Rev. Lett.* 49:1565–1568.

42. Zhao, X., G. Bartolucci, …, C. A. Weber. 2021. Thermodynamics of wetting, prewetting and surface phase transitions with surface binding. *New J. Phys.* 23:123003.

43. Rouches, M., S. L. Veatch, and B. B. Machta. 2021. Surface densities prewet a near-critical membrane. *Proc. Natl. Acad. Sci. USA.* 118:e2103401118.

44. Weakly, H. M. J., and S. L. Keller. 2024. Coupling liquid phases in 3D condensates and 2D membranes: Successes, challenges, and tools. *Biophys. J.* 123:1329–1341.

45. Case, L. B., M. De Pasquale, …, M. K. Rosen. 2022. Synergistic phase separation of two pathways promotes integrin clustering and nascent adhesion formation. *Elife.* 11:e72588.

46. Litschel, T., C. F. Kelley, …, P. Schwille. 2024. Membrane-induced 2D phase separation of the focal adhesion protein talin. *Nat. Commun.* 15:4986.

47. Harayama, T., and H. Riezman. 2018. Understanding the diversity of membrane lipid composition. *Nat. Rev. Mol. Cell Biol.* 19:281–296.

48. Orre, T., A. Joly, …, G. Giannone. 2021. Molecular motion and tridimensional nanoscale localization of kindlin control integrin activation in focal adhesions. *Nat. Commun.* 12:3104.

49. Cain, R. J., and A. J. Ridley. 2009. Phosphoinositide 3-kinases in cell migration. *Biol. Cell.* 101:13–29.

50. Iijima, M., Y. E. Huang, and P. Devreotes. 2002. Temporal and spatial regulation of chemotaxis. *Dev. Cell.* 3:469–478.

51. Karunarathne, W. K. A., L. Giri, …, N. Gautam. 2013. Optical control demonstrates switch-like PIP3 dynamics underlying the initiation of immune cell migration. *Proc. Natl. Acad. Sci. USA.* 110:E1575–E1583.

52. Carnero, A., C. Blanco-Aparicio, …, J. F. M. Leal. 2008. The PTEN/PI3K/AKT signalling pathway in cancer, therapeutic implications. *Curr. Cancer Drug Targets.* 8:187–198.

53. Di-Luoffo, M., Z. Ben-Meriem, …, J. Guillermet-Guibert. 2021. PI3K functions as a hub in mechanotransduction. *Trends Biochem. Sci.* 46:878–888.

54. Norman, L. L., R. J. Oetama, …, H. Aranda-Espinoza. 2010. Modification of Cellular Cholesterol Content Affects Traction Force, Adhesion and Cell Spreading. *Cell. Mol. Bioeng.* 3:151–162.

55. Eich, C., C. Manzo, …, A. Cambi. 2016. Changes in membrane sphingolipid composition modulate dynamics and adhesion of integrin nanoclusters. *Sci. Rep.* 6:20693.

Biophysical Journal, Volume *125*

Supplemental information

Synergistic effect of PIP_2 and PIP_3 on membrane-induced phase separation of integrin complexes

Chiao-Peng Hsu, Arsenii Hordeichyk, Jonas Aretz, Reinhard Fässler, and Andreas R. Bausch

Supplementary Material for Synergistic effect of PIP_2 and PIP_3 on membrane-induced phase separation of integrin complexes

Chiao-Peng Hsu,[1] Arsenii Hordeichyk,[1,2] Jonas Aretz,[3] Reinhard Fssler,[3] and Andreas R. Bausch[1,2,*]

[1] *Chair for Cellular Biophysics, Center for Functional Protein Assemblies, Center for Organoid Systems, Department of Bioscience, Technical University of Munich, Technical University of Munich School of Natural Sciences, Garching 85748, Germany*

[2] *Max Planck School Matter to Life, Jahnstrae 29, D-69120 Heidelberg, Germany*

[3] *Department of Molecular Medicine, Max Planck Institute of Biochemistry, Martinsried 82152, Germany*

CO-SEDIMENTATION ASSAY

The protein-lipid co-sedimentation assay is based on a reported protocol [1]. Lipid mixtures of 5% PIP_2 (94.5 mol% DOPC, 5 mol% PI(4,5)P_2, and 0.5 mol% PEG2000-PE), 5% PIP_3 (94.5 mol% DOPC, 5 mol% PI(3, 4,5)P_3, and 0.5 mol% PEG2000-PE) were mixed in chloroform. Lipid films were prepared by drying the lipid mixtures under a stream of nitrogen and placed under a vacuum for at least 2 h. Lipid films were hydrated and re-suspended by vortexing for 30 min in HEPES buffer (pH 7.4, 50 mM HEPES, and 150 mM KCl) at a final lipid concentration of 1 mM. The obtained PIP_2- and PIP_3-liposomes were used within 2 h.

Protein aggregates were removed by centrifugation (436000 rcf, 30 min) at 4 °C. Protein concentrations were determined with a UV-Vis spectrometer. Combinations of kindlin/talin with PIP_2-/PIP_3-liposomes and kindlin/talin alone (negative controls) were mixed in HEPES buffer with a final concentration of 1 μM of protein, 0.5 mM of liposome, and 1 mM DTT in a volume of 50 μL. The solutions were incubated for 30 min at room temperature.

After 30-min incubation, the solutions were ultra-centrifuged at 436000 $\times$ g for 30 min at 4 °C. The supernatant was removed, and the pellet was re-suspended in 50 μL of HEPES buffer. Samples were analyzed with SDS-PAGE. The protein-bound fraction was estimated from the measured intensities from the gel images:

$$\text{Protein-bound fraction} = \frac{\text{Intensity}_{\text{Pellet}}}{\text{Intensity}_{\text{Pellet}} + \text{Intensity}_{\text{Supernatant}}}$$

PROTEIN LABELING

Atto 565-NHS-ester (Sigma-Aldrich) was dissolved in DMSO (Sigma-Aldrich) at the concentration of 5 μg/μL. Kindlin was re-buffered to phosphate-buffered saline using Zeba^{TM} Spin Desalting Columns (7K, 0.5 mL; Thermo Scientific) and mixed with the Atto 565-NHS-ester solution in a mole ratio of 1:2.5. The mixture was kept for 2 h in room temperature. After the 2-h reaction, the mixture was re-buffered to tris-buffered saline to quench the reaction and remove the excess dye. The labeled proteins were stored at -80 °C.

CO-LOCALIZATION EXPERIMENT

The sequential assembly of 1 μM kindlin and 1 μM talin followed by 200 nM paxillin and 100 nM FAK on β1-bound 5% PIP_2, 5% PIP_3, and 2.5% PIP_2+2.5% PIP_2 membranes were performed as described in the manuscript. 5 mol% labeled kindlin was used to visualize the condensates on the membranes (Figure S6).

---

* abausch@mytum.de

[1] Senju, Y., Lappalainen, P. & Zhao, H. *Liposome Co-sedimentation and Co-flotation Assays to Study LipidProtein Interactions*, 195–204 (Springer US, New York, NY, 2021).

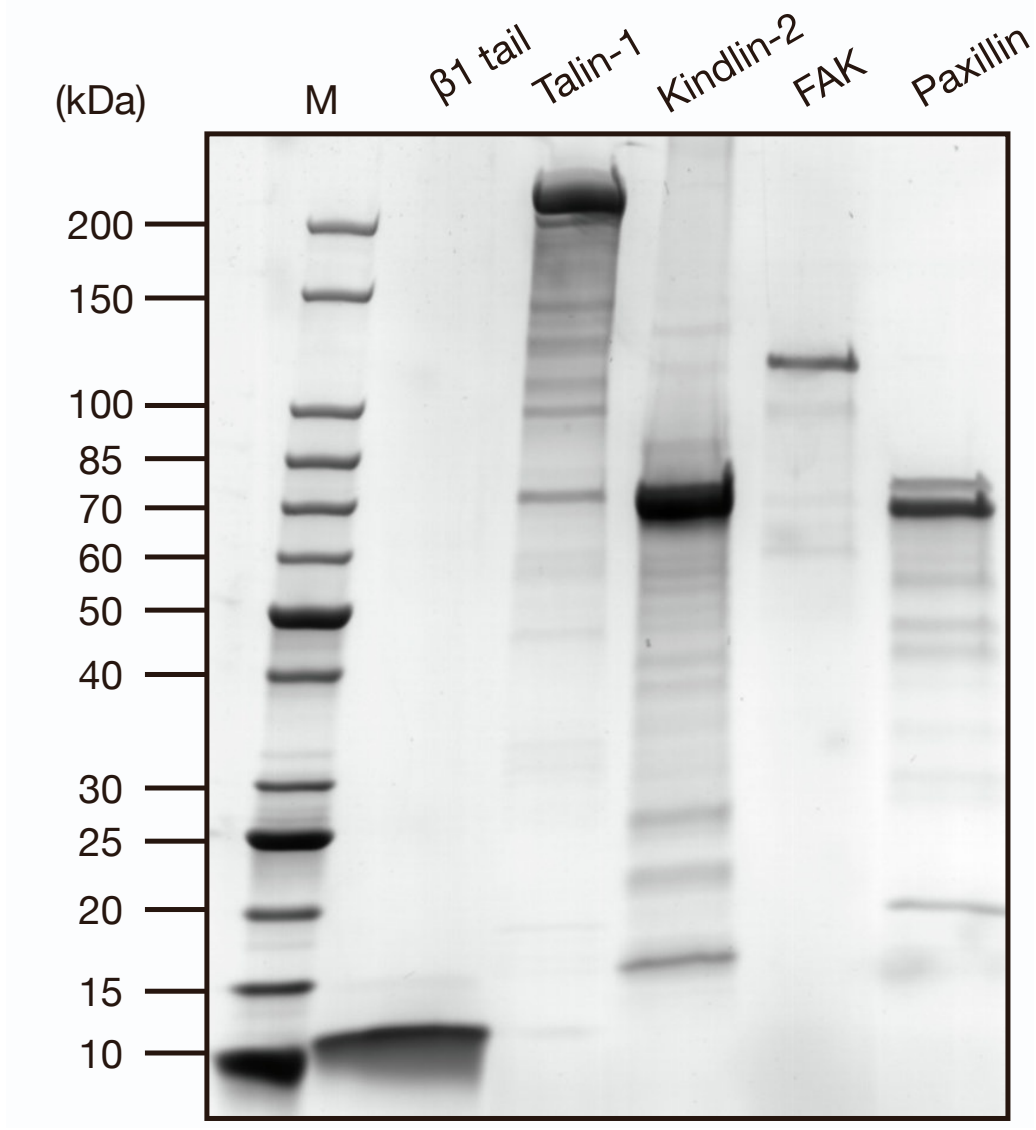

Supplementary Figure 1. **Protein characterization.** SDS-PAGE gel of purified proteins stained with Quick Coomassie.

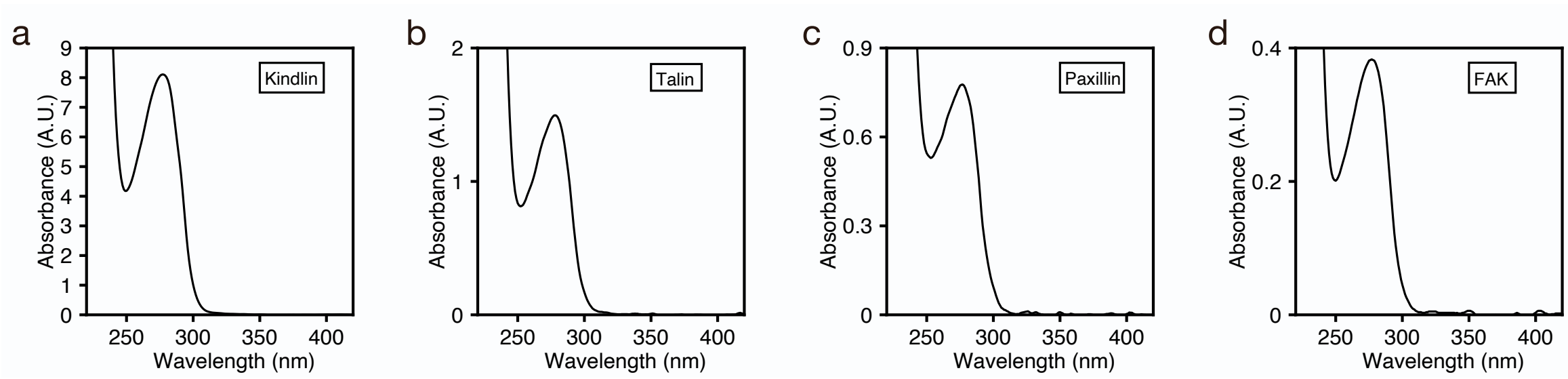

Supplementary Figure 2. **Protein characterization.** UV-Vis spectrums of kindlin (a), talin (b), paxillin (c), and FAK (d).

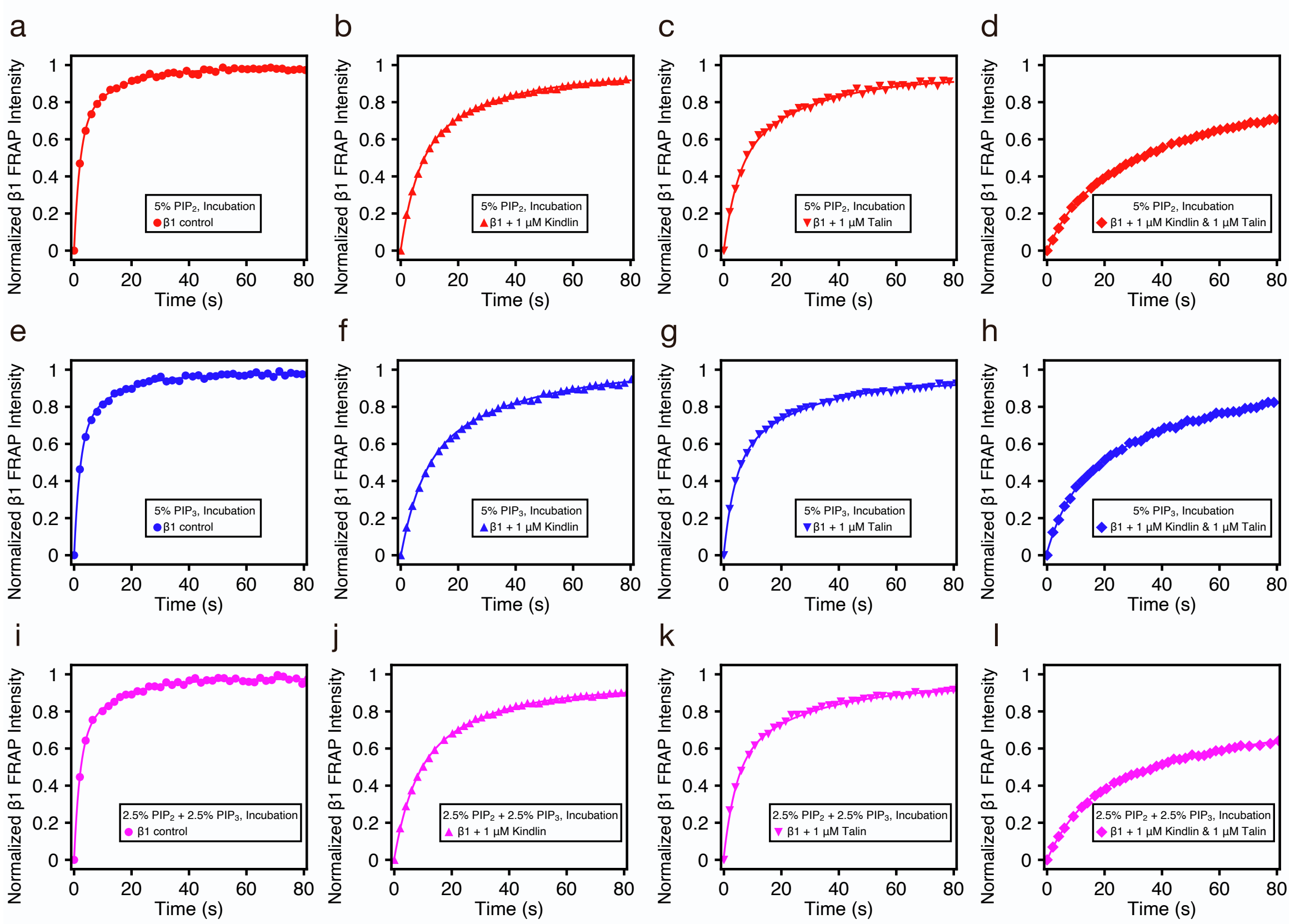

Supplementary Figure 3. **Diffusion of $\beta 1$ on membranes.** Normalized FRAP measurements of $\beta 1$ with 5% PIP_2 (a–d), 5% PIP_3 (e–h), and 2.5% PIP_2+2.5% PIP_2 (i–l) membranes. Solid curves show the double component exponential recovery fittings.

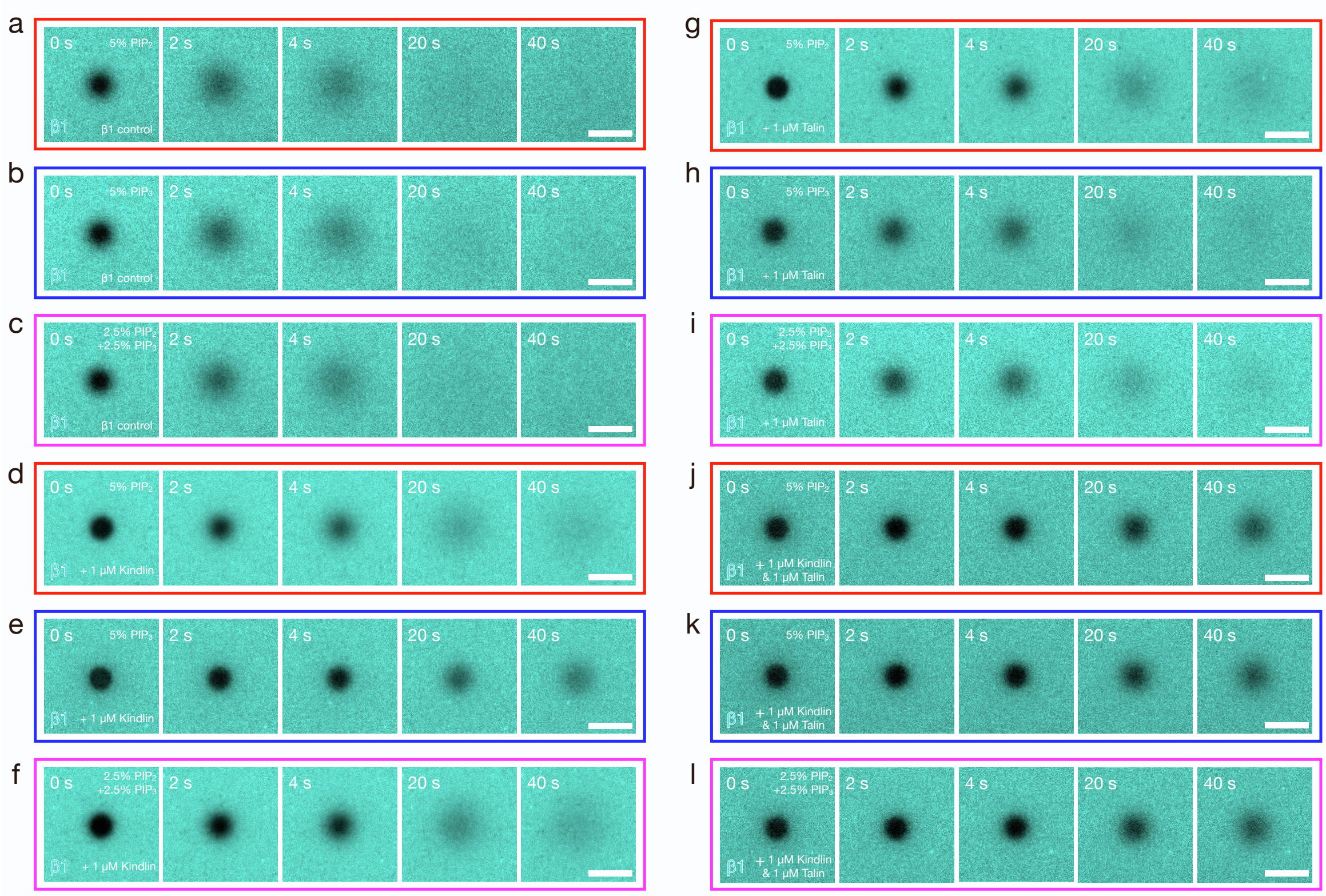

Supplementary Figure 4. **Diffusion of $\beta 1$ on membranes.** FRAP images of $\beta 1$ on 5% PIP_2 (a), 5% PIP_3 (b), and 2.5% PIP_2+2.5% PIP_2 (c) membranes. FRAP images of $\beta 1$ after 30 min incubation with 1 μM kindlin on 5% PIP_2 (d), 5% PIP_3 (e), and 2.5% PIP_2+2.5% PIP_2 (f) membranes. FRAP images of $\beta 1$ after 30 min incubation with 1 μM talin on 5% PIP_2 (g), 5% PIP_3 (h), and 2.5% PIP_2+2.5% PIP_2 (i) membranes. FRAP images of $\beta 1$ after 30 min incubation with 1 μM kindlin and 1 μM talin on 5% PIP_2 (j), 5% PIP_3 (k), and 2.5% PIP_2+2.5% PIP_2 (l) membranes.

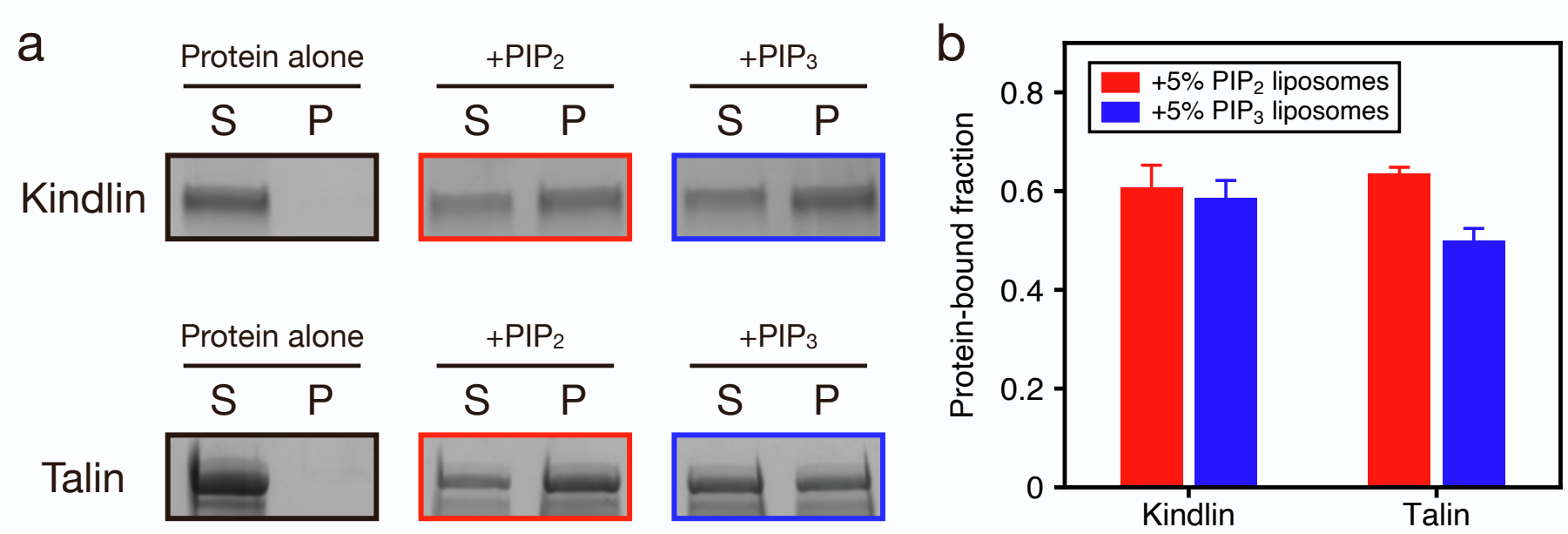

Supplementary Figure 5. **Interactions between kindlin/talin and PIP_2/PIP_3.** (a) Images of SDS-PAGE gels stained with Quick Coomassie after the PIP_2- /PIP_3-liposome co-sedimentation assay of kindlin (up) and talin (bottom). (b) Fraction of kindlin or talin bound to the PIP_2-liposomes (red) and PIP_2-liposomes (blue). Error bars represent the standard deviations from 3 measurements.

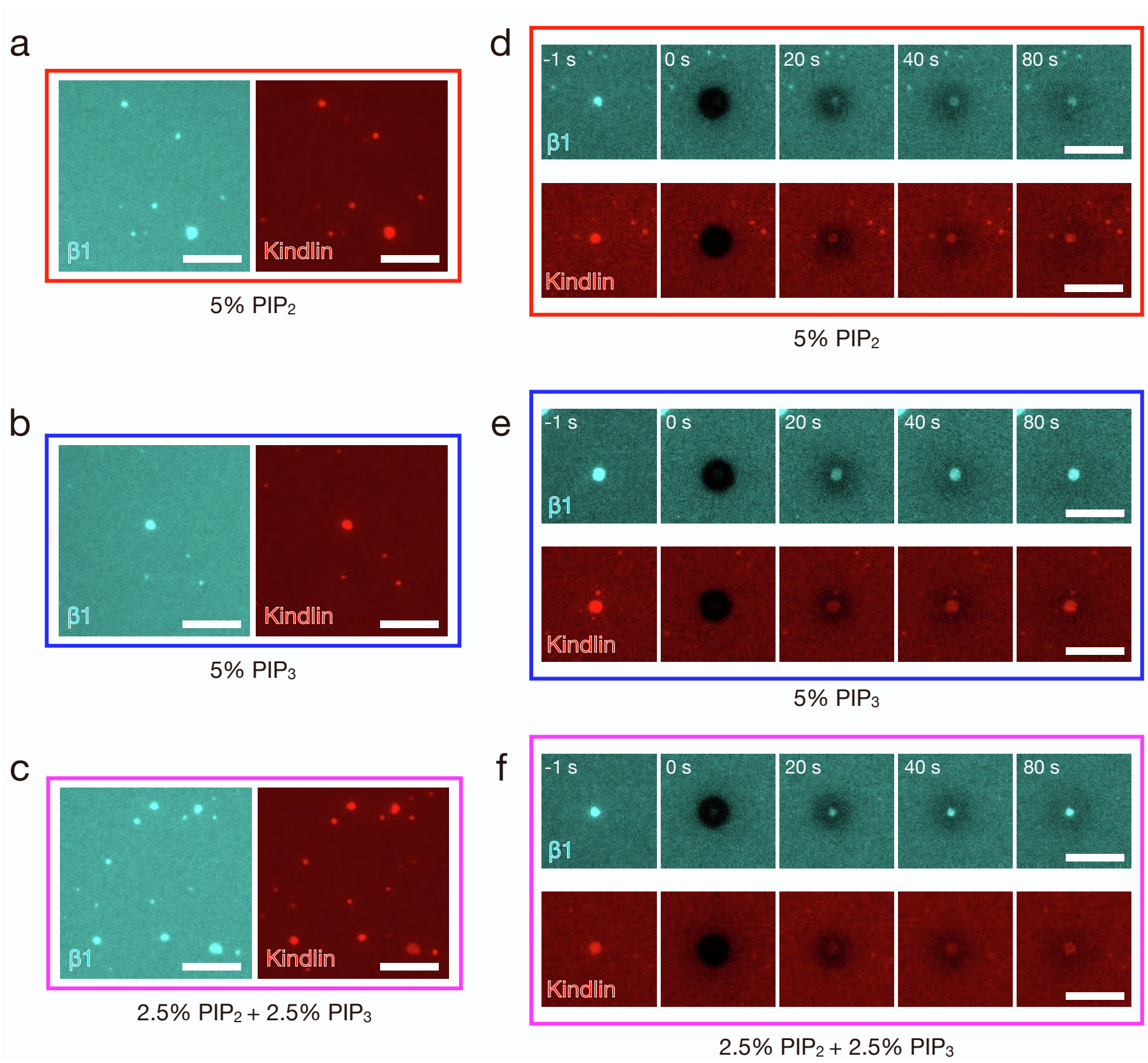

Supplementary Figure 6. **Emergence of $\beta1$ clusters.** $\beta1$ and kindlin clusters formed after sequential incubation on 5% PIP_2 (a), 5% PIP_3 (b), and 2.5% PIP_2+2.5% PIP_3 (c) membranes. FRAP images of a $\beta1$ cluster and a kindlin cluster formed on 5% PIP_2 (d), 5% PIP_3 (e), and 2.5% PIP_2+2.5% PIP_3 membranes (f). Scale bars, 10 μm.

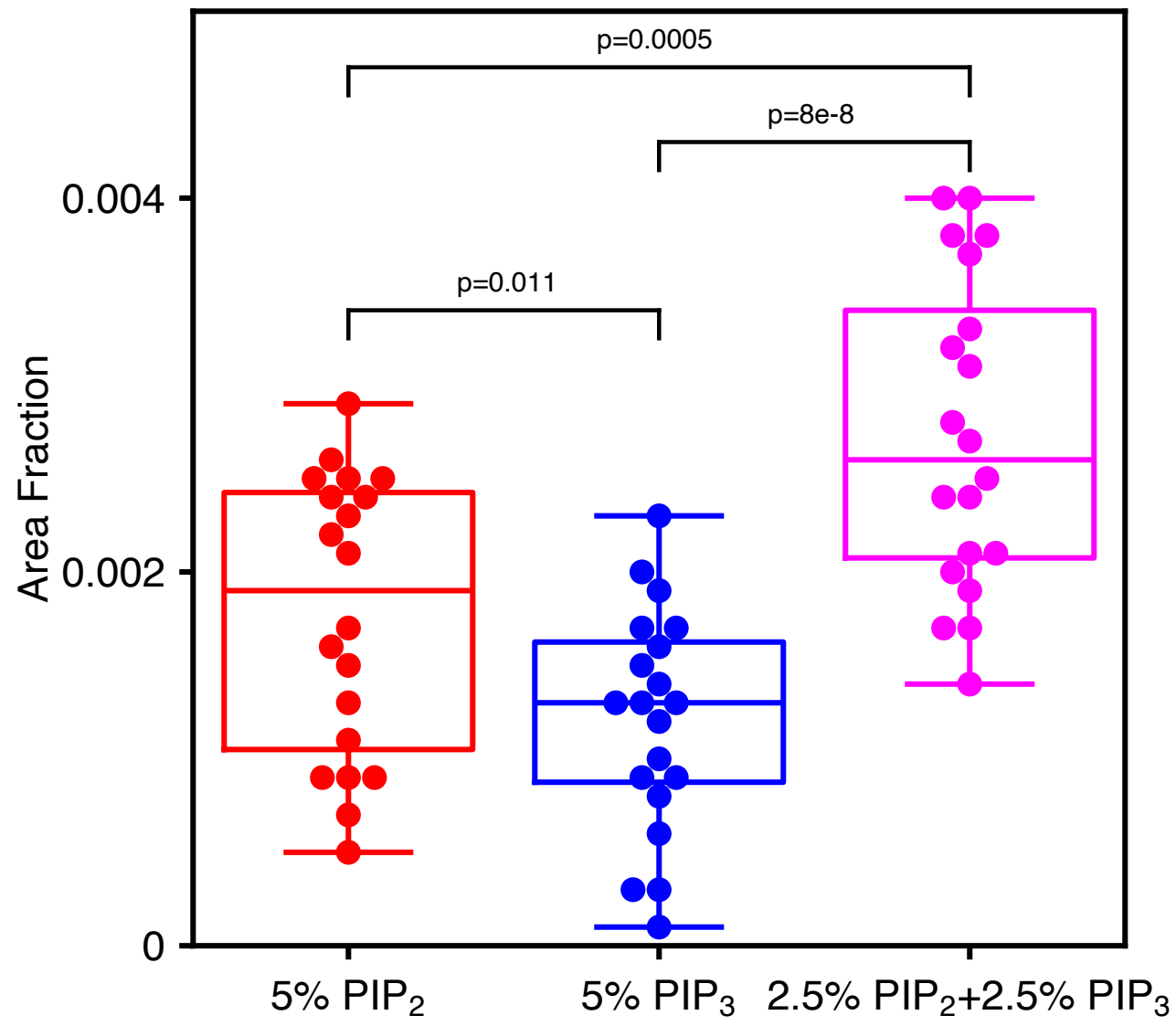

Supplementary Figure 7. **Area fraction of β1 clusters.** Area fraction of β1 clusters formed after 90-min incubation on 5% PIP_2, 5% PIP_3, and 2.5% PIP_2+2.5% PIP_2 membranes. Each condition contains 20 measurements from 4 independent experiments. Box plots indicate the median (line); 25th and 75th percentile (box); 0th and 100th percentile (whiskers). Significance tested with unpaired t-test.

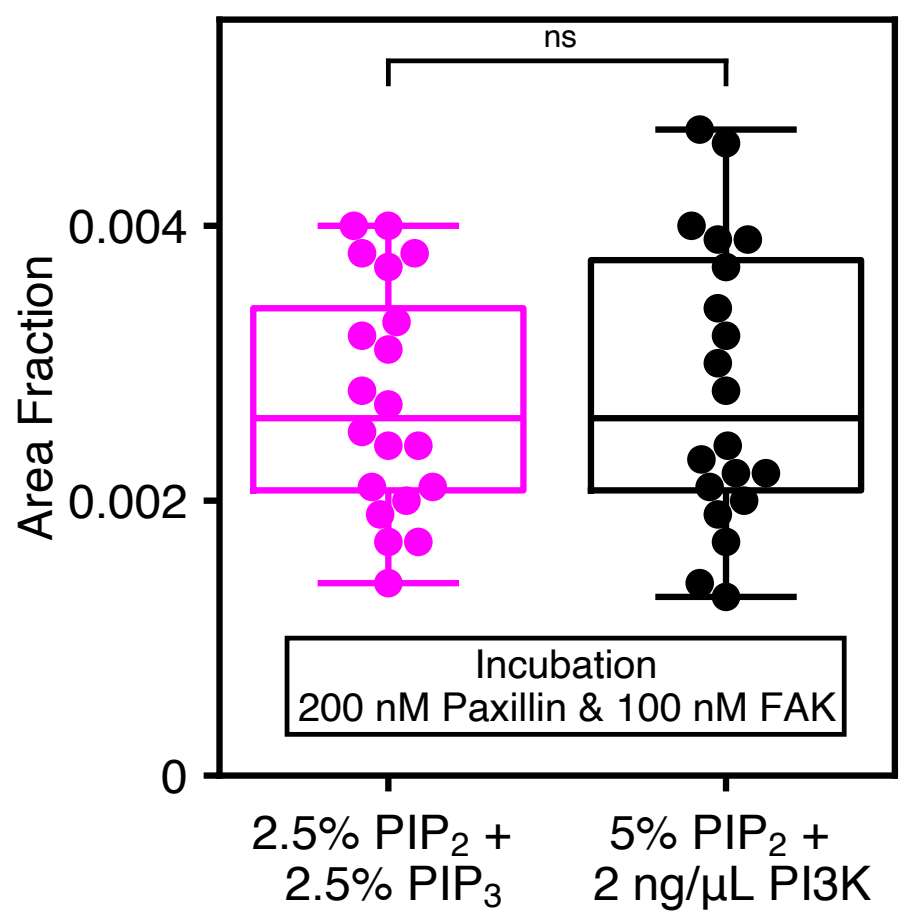

Supplementary Figure 8. **Area fraction of β1 clusters.** Area fraction of β1 clusters formed after 90-min incubation (200 nM paxillin and 100 nM FAK) on 2.5% PIP_2+2.5% PIP_2 membranes and 5% PIP_2 membranes with 2 ng/μL PI3K. Each condition contains 20 measurements from 4 independent experiments. Box plots indicate the median (line); 25th and 75th percentile (box); 0th and 100th percentile (whiskers). Significance tested with unpaired t-test.

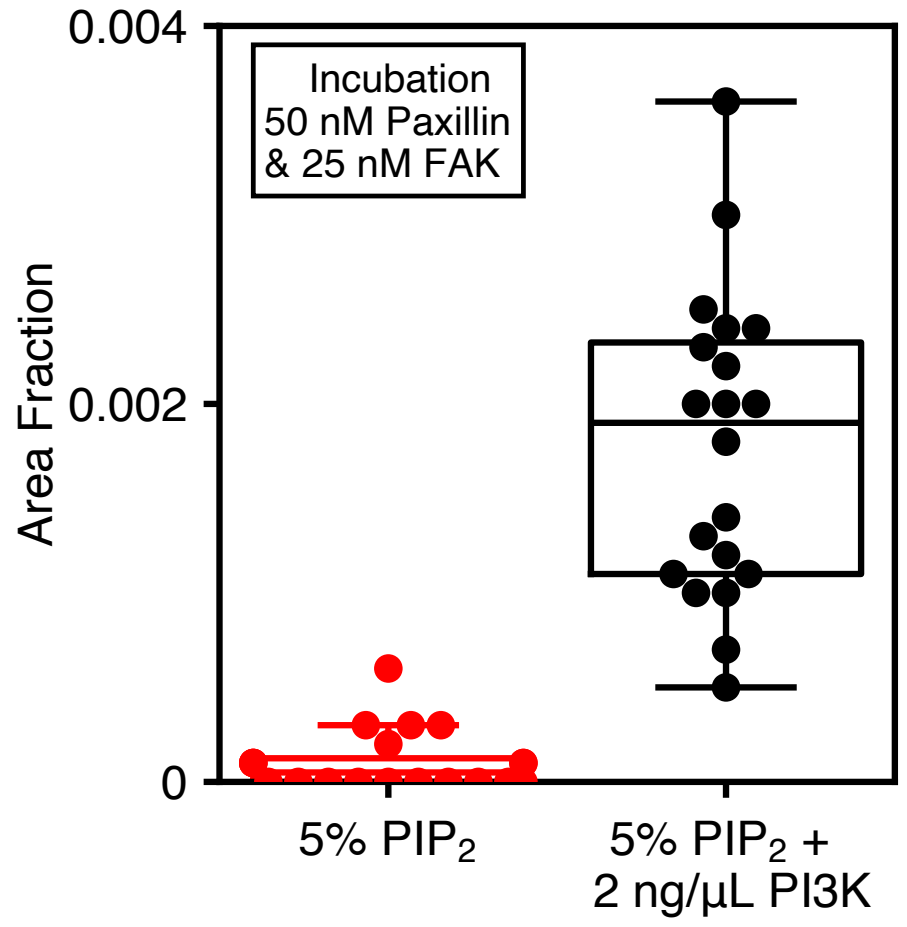

Supplementary Figure 9. **Area fraction of β1 clusters.** Area fraction of β1 clusters formed after 90-min incubation (50 nM paxillin and 25 nM FAK) on 5% PIP_2 membranes and 5% PIP_2 membranes with 2 ng/μL PI3K. Each condition contains 20 measurements from 4 independent experiments. Box plots indicate the median (line); 25th and 75th percentile (box); 0th and 100th percentile (whiskers).
